# Futureproofing the healthcare workforce in Europe: understanding and addressing psychological distress and occupational outcomes

**DOI:** 10.1016/j.lanepe.2025.101463

**Published:** 2025-10-06

**Authors:** Pamela Almeida-Meza, Sarah Ledden, Brendan Dempsey, Alexandria Smith, Bethany Croak, Rupa Bhundia, Danielle Lamb, Aiysha Malik, Rosalind Raine, Cassie Redlich, Simon Wessely, Sharon Stevelink, Neil Greenberg

**Affiliations:** a Department of Psychological Medicine, Institute of Psychiatry, Psychology & Neuroscience, King's College London, London, UK; b Department of Primary Care and Population Health, University College London, London, UK; c World Health Organization Regional Office for Europe, Geneva, Switzerland; d World Health Organization Regional Office for Europe, Copenhagen, Denmark

**Keywords:** Healthcare workers, Work-related stress, Psychological distress, Workforce retention, Burnout

## Abstract

Healthcare systems across Europe face a workforce crisis characterised by staff shortages, high turnover, and psychological distress among healthcare workers (HCWs). To understand the true scope and extent of psychological distress and occupational outcomes among HCWs and key areas to target for actionable solutions to this, a comprehensive overview of the evidence is needed. In this Series paper, we have synthesised findings from existing systematic reviews and meta-analyses to assess the prevalence of psychological distress among HCWs in Europe, examine associated risk and protective factors, and evaluate the effectiveness of interventions targeting these. The Series paper shows that HCWs experience high levels of work-related psychological distress, particularly burnout. Organisational factors, such as staff shortages, high workloads, and poor leadership, are key contributors to HCWs psychological distress. Organisational-level, rather than individual-level, interventions showed greater promise in reducing psychological distress and improving occupational outcomes. Perhaps surprisingly, currently published reviews did not focus on diagnosable mental disorders, instead relying on self-report symptom-based measures, likely overestimating prevalence. Our findings show limited research available on psychological distress and occupational outcomes among non-clinical staff, HCWs from ethnic minorities, and countries outside Western Europe. We recommend long-term investment in fostering safe and supportive workplaces and implementing evidence based multi-level mental health initiatives that are co-produced with HCWs. We also advocate for high quality, longitudinal research and policies that prioritise protecting the mental health and occupational safety of HCWs in order to safeguard their wellbeing and futureproof healthcare system sustainability.

## Introduction

Healthcare systems worldwide are experiencing a mounting workforce crisis with difficulties in recruiting and retaining the healthcare workers (HCWs) necessary to provide adequate healthcare delivery.^1^ The reasons for this are complex and are also regionally specific.^2^^,^^3^ In 2006, the World Health Organisation (WHO) estimated that there was a global deficit of 4.3 million HCWs, particularly in lower-income countries.^4^ By 2020, this deficit had increased to an estimated 15 million, highlighting the urgency of addressing this issue.^5^ Increasingly, HCWs are taking periods of sickness absence or leaving their professions, frequently citing mental health concerns, stress, and burnout stemming from multiple occupational stressors.^6^ Among those who remain, performance often declines as they contend with increasing healthcare system demands, driven by higher patient acuity and shrinking workforce, alongside diminished occupational support and deteriorating working conditions.^7^ While not unique to Europe, the region exemplifies the global challenge of an ageing health workforce and increasing demand due to population demographic changes. Europe faces a workforce shortage of approximately 1.2 million HCWs, with a substantial portion of the healthcare workforce nearing retirement, in tandem with an ageing population, which places additional strain on the healthcare systems.^1^^,^^8^ Many countries in the region report low graduate replacement rates and continued challenges with retention.^1^ These trends reflect broader concerns related to occupational outcomes among this population, including low job satisfaction, high turnover, turnover intention, absenteeism, and presenteeism. The COVID-19 pandemic exacerbated pressures on already strained healthcare systems, further compromising workforce health and safety and accelerating attrition of critical personnel.^9^ As the backbone of healthcare delivery, protecting mental health and promoting recruitment and retention of HCWs are a public health imperative.

**Key messages**

• Psychological distress is widely reported among European healthcare workers (HCWs)
• Burnout is the most commonly measured outcome
• Organisational factors such as staff shortages, high workloads and poor leadership, are key contributors to HCWs psychological distress. Nurses and HCWs from ethnic minorities are disproportionately affected
• Workplace stressors are associated with negative psychological distress and occupational outcomes
• Psychological distress, whether work-related or associated with mental disorders, contributes to low job satisfaction and high turnover intention
• Organisational-level interventions show greater promise than individual-level interventions in reducing psychological distress by targeting systemic problems such as workload, staffing, support, and leadership issues
• There is limited high-quality research on mental disorders, non-clinical HCWs, and European regions outside Western Europe. Measurement inconsistencies restrict comparability across studies
• Addressing modifiable workplace risk factors through policy, leadership and investment is key to protecting HCWs psychological distress and ensuring an effective and sustainable healthcare workforce

**Search strategy and selection criteria**

References for this umbrella review were identified through systematic searches of six electronic databases (Cochrane Library, Medline, PsycINFO, Embase, AMED, and CINAHL). To ensure relevance to contemporary healthcare contexts, we restricted to studies published between 2010 and 2024. The search strategy combined MeSH terms and keywords related to healthcare personnel (e.g., “nurse”, “physician”, “medical staff”), mental disorders (e.g., “mental disorders”, “depression”, “burnout”), and occupational outcomes (e.g., “retention”, “satisfaction”). Boolean operators and database-specific filters were applied to limit results to studies conducted in Europe. We included peer-reviewed empirical reviews, such as systematic reviews, meta-analyses, scoping and rapid reviews, reporting on clinical staff and non-clinical staff. Studies that included data from non-European regions were eligible if findings from Europe were also included. Publications not available in English, focused exclusively on students or social care workers, and published in non-empirical formats (e.g., opinion pieces and letters) were excluded. The complete search strategy is available in Supplementary Table S1. Details of reviews retrieved from searches, including quality appraisal ratings, are provided in Supplementary Table S2.

In this review, we consolidated the current evidence on workplace stressors, psychological distress and mental disorders and their impact on turnover and retention among HCWs across Europe. This evidence provides a foundation for targeted interventions and policy at organisational and governmental levels that address key stressors, potentially reducing mental disorders or related symptomatology, boosting recruitment and retention, and ultimately enhancing patient safety through a more stable and healthier workforce. Our review is structured around three objectives related to the mental health of HCWs in Europe. Objective 1: Examine the current prevalence of mental disorders among HCWs; Objective 2: Investigate the factors that exacerbate or mitigate these risks; Objective 3: Assess the existing evidence on interventions aimed at preventing or addressing mental disorders and adverse occupational outcomes amongst HCWs.

## Mental disorders in HCWs: what is being measured?

**Fig. 1:**
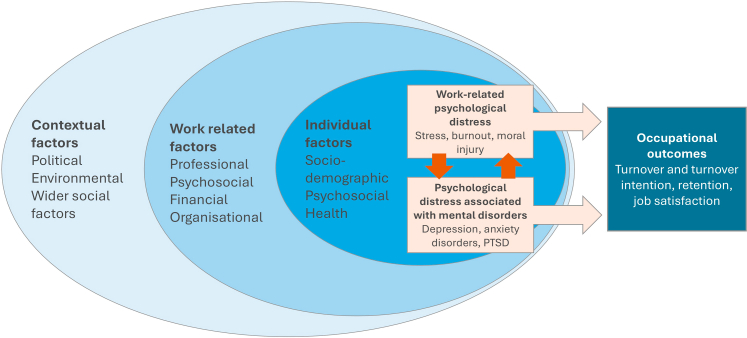
**Ecological model of multi-level influences on psychological distress and occupational outcomes**.

Few studies included in this review explored mental disorders. Among those that did, none conducted meta-analysis to estimate the prevalence of such conditions. Most reviews included studies approximating disorders using self-report screening. Furthermore, a wide range of overlapping and variably defined psychological outcomes were reported across individual reviews. These included terms such as stress, burnout, fatigue, anxiety, low morale, and dissatisfaction, as well as broader terms such as mental health problems, psychiatric symptoms, and psychological distress. In light of this heterogeneity in terminology and measurement, we used ‘psychological distress’ as an overarching term throughout our review to describe this spectrum of self-reported outcomes. Within this, we distinguish between distress associated with mental disorders (e.g., anxious, depressive, and trauma related symptoms), and work-related psychological distress (e.g., stress, burnout, and moral distress). While these outcomes vary in their clinical recognition and definitions, they collectively reflect the psychological impact of occupational exposure and stressors among HCWs. Importantly, these two domains of psychological distress are likely to be interdependent whereby distress associated with work-related stressors may increase the vulnerability of developing distress related to mental disorders and vice versa (see Fig. 1).

## Psychological distress among healthcare workers

Few reviews examined the prevalence of distress associated with mental disorders. A meta-analysis found nurses globally experience moderate secondary traumatic stress.^10^ A scoping review on maternity staff worldwide reported that COVID-19-related challenges contributed to anxiety symptoms, depression, and PTSD.^11^ A narrative review cited survey findings showing 92% of UK emergency service staff had experienced poor mental health, and 62% had received treatment.^12^ In terms of suicide risk, one review found 27% of UK emergency staff, including ambulance workers, had experienced stress-related suicidal ideation^12^ and a meta-analysis showed female physicians had a higher suicide risk than the general population (mean estimate: 1.76, 95% CI: 1.40–2.21), while no difference was found for male physicians.^13^

Most of the available evidence on psychological distress among HCWs focused on work-related psychological distress outcomes such as burnout and moral distress. Burnout was by far the most frequently reported outcome.^10^^,^^14^, ^15^, ^16^, ^17^, ^18^, ^19^, ^20^, ^21^, ^22^, ^23^, ^24^, ^25^ Although burnout is not considered a mental disorder, it is recognised as a psychological phenomenon that results from chronic work-related stress^26^ and has been associated with mental disorder and occupational outcomes.^27^ The reported burnout prevalence varies widely depending on speciality, from 8% among physiotherapists^14^ to 32% among nurses^25^ and 49% among physicians.^18^ Narrative reviews have suggested that burnout is particularly elevated in specific clinical contexts such as emergency departments (57%),^23^ paediatric intensive care units (79%),^22^ and palliative care (17%).^20^ However, burnout prevalence estimates have also been found to vary widely within professional settings, with figures ranging from 3 to 65% among oncology nurses,^19^ 9 to 68% among urologists,^21^ and 3 to 72% among physicians.^28^ These inconsistencies and variability in reported prevalence rates have been attributed to differences in measurement, including the use of non-standardised thresholds in widely used tools such as the Maslach Burnout Inventory (MBI)^29^ and Professional Quality of Life scale^30^ (see Table 1). The most commonly used versions of the MBI include 22 items assessing burnout across three dimensions: emotional exhaustion, depersonalisation, and personal accomplishment. However, studies vary widely in the cut-off scores used for each subscale. For example, the threshold for depersonalisation ranges from ≥10 to ≥14, and for personal accomplishment either ≥31 or ≥33 is used. Furthermore, a frequently used criterion to define overall burnout is meeting the cut-off on all three subscales, but some studies apply alternative combinations.

**Table 1:** Overview of burnout prevalence from meta-analyses in European healthcare workers, highlighting variability across occupational groups and MBI and ProQoL subscales.

Occupational group (n)	Maslach Burnout Inventory											
	Total score			EE subscale			DP subscale			PA subscale		
	%	95% CI		%	95% CI		%	95% CI		%	95% CI	
Physicians^18^ (n = 9667)^a^	49	45	53	–	–	–	–	–	–	–	–	–
Dentists^15^ (n = 4327)^b^	17	5	33	–	–	–	–	–	–	–	–	–
Nurses^25^ (n = 14,690)^b^	32	22	43	–	–	–	–	–	–	–	–	–
Physiotherapists^14^ (n = 5984)	8	4	15	27	21	34	23	15	32	25	15	40
GPs^16^ (n = 6451)^b^	–	–	–	35	28	43	29	25	32	25	20	31
Oncologists^17^ (n = 1585)^b^	–	–	–	32	26	38	30	22	39	22	15	30
Oncology nurses^17^ (n = 2293)^b^	–	–	–	28	23	33	20	15	26	20	13	29
Oncology nurses^24^ (n = 771)^b^	–	–	–	20	18	22	6	4	7	31	23	40

Occupational group (n)	Professional Quality of Life scale											
	Total score			CS			Burnout			STS		
	%	95% CI		%	95% CI		%	95% CI		%	95% CI	
Nurses^10^ (n = 382)^b^	–	–	–	32	27	37	24	20	27	20	16	24

MBI = Maslach Burnout Inventory^29^: comprised of three subscales, where higher scores in the Emotional Exhaustion (EE) and Depersonalisation (DP) sub-scales indicate greater burnout, while higher scores in the Personal Accomplishment (PA) sub-scale reflect lower burnout. The most used version of the MBI includes 22-items. There was substantial variation in the cut-off scores used for the subscales. Common cut-off were ≥27 for EE, ≥10–14 for DP, and ≤31–33 for PA. A frequently used approach to define overall burnout was meeting the cut-off for all three subscales, although some studies applied alternative combinations or criteria.

ProQoL = Professional Quality of Life scale^30^; comprised of three subscales with higher scores in the Burnout and Secondary Traumatic Stress (STS) subscales indicating greater occupational stress, while higher scores on the Compassion Satisfaction (CS) subscale suggest more positive professional quality of life. Each sub-scale is calculated by aggregating the 10 items, yielding possible scores ranging from 10 to 50. Scores of ≤22 indicate low levels, scores between 23 and 41 indicate moderate levels, and scores ≥42 reflect high levels.

% = Percentage of healthcare workers meeting cut-off score on that scale, indicating probable burnout; 95% CI = 95% Confidence Intervals.

a This review only included research from European countries.

b All other reviews included research from European and non-European countries, though these also presented sub-group analysis of estimated burnout values in Europe (which are presented).

Estimates of burnout prevalence among HCWs in Europe were inconsistent across reviews, with three suggesting higher rates in European settings compared to other regions,^15^, ^16^, ^17^ two indicating lower rates,^10^^,^^24^ and one reporting no significant regional differences.^25^ Although these inconsistencies largely reflect methodological differences in threshold selection, other sources of bias should be considered when assessing burnout. For instance, cross-national comparisons should be interpreted with caution since cultural differences influence how work-related stress is perceived and internalised.^31^ Moreover, although some comparative data suggests higher burnout among HCWs than other occupational groups (e.g., 40% vs 25% in the USA and 18% vs 17% in Switzerland^32^^,^^33^), high levels of burnout reported in occupational settings may in part be influenced by contextual factors and reporting biases leading to overestimation of true prevalence rates.^34^

Moral distress is reported as stemming from someone's inability to take ethically appropriate actions due to institutional or systemic constraints and is marked by perceived violation of personal and professional integrity.^35^ In line with this, a scoping review found that being forced to act against evidence, professional recommendations, or their ethical and moral values and beliefs to provide care during the COVID-19 pandemic led to psychological distress amongst maternity staff.^11^ Like burnout, moral distress is not a formal mental disorder, but it is increasingly recognised as an important construct in the context of healthcare work, with evidence linking it to mental disorders and occupational outcomes.^36^, ^37^, ^38^ For example, amongst paediatric nurses, moral distress has been associated with depression, anxiety symptoms, anger, and sadness.^39^ Related concepts, such as moral injury, have also been shown to be prevalent among UK HCWs, particularly in the form of betrayal (i.e., feeling abandoned or unsupported by leadership or the public), and are associated with significantly increased risk of PTSD, mental disorders, and burnout.^40^ However, moral distress is a relatively new concept in research, and current evidence is limited by conceptual inconsistencies and reliance on self-reported measures, which might not fully capture the complexity of the experience.^35^^,^^41^ Thus, although recent meta-analytic data indicated low levels of moral distress among HCWs,^42^ conflicting evidence exists^40^^,^^43^ and further high-quality research is needed to better understand the true burden and implications of moral distress among HCWs.

The lack of reviews on diagnosable mental disorders restricts our understanding of these and the contribution of occupational stressors to them. More high-quality research is needed in Europe using gold-standard diagnostic measures, rather than symptom screening tools which tend to overestimate prevalence. However, collecting diagnostic data is highly labour-intensive and costly, requiring researchers to be trained to a sufficient standard. Studies should also employ rigorous recruitment methods, including oversampling underrepresented groups, to improve the generalisability of findings, and greater standardisation of outcome measurement is needed to facilitate comparisons across studies. Ultimately, researchers must strike a balance between timeliness, cost, and methodological rigour to ensure efficient and valid contributions to the literature.

## Factors that exacerbate or mitigate risks to HCW psychological distress and occupational outcomes

HCWs psychological distress and the associated occupational outcomes are a result of dynamic, reciprocal interactions between the individual and multiple levels of their environment, ranging from direct work settings to broader societal structures. Based on available evidence, we developed a conceptual framework influenced by the ecological model of health promotion^44^ and Karasek and Theorell's demand-control theory of job stress^45^ to illustrate the relationship between workplace stress, mental disorders, and occupational outcomes among HCWs, identifying the pathways through which different factors influence these (Fig. 1).

### Profession and setting

Studies involving emergency physicians, ambulance staff, forensic professionals, oncologists, oncology nurses, and paediatric nurses suggested that these professionals face increased physical and emotional demands compared to other specialities.^12^^,^^17^^,^^18^^,^^39^^,^^46^ These roles involve high paced work, ethically challenging decisions, handling challenging cases (e.g., performing autopsies or taking care of critically ill children) and delivering upsetting information to families. This was associated with secondary trauma, PTSD, burnout, and moral distress.^12^^,^^17^^,^^39^^,^^46^ Amongst HCWs, nurses have consistently been found to report a higher prevalence of burnout.^22^^,^^25^ Nurses, especially those working in emotionally demanding contexts (i.e., palliative care, obstetrics, paediatrics, emergency, intensive care unit) report higher stress, moral distress, burnout, and severe compassion fatigue,^10^^,^^20^^,^^22^^,^^25^^,^^42^ with one study suggesting that these outcomes might be partly due to increased exposure to patient-related violence.^47^ Some HCWs find the emotional burden of healthcare work scenarios challenging^48^; particularly when frequently exposed to death and suffering.^49^^,^^50^ These have been associated with high levels of stress, burnout, and moral distress,^11^^,^^22^^,^^50^ with the latter being predictive of staff intention to leave or turnover (Panel 1).^50^^,^^56^

Panel 1 **Psychological distress during the COVID-19 pandemic.**

Nurses appeared to be the most affected group of HCWs during the COVID-19 pandemic reporting elevated psychological distress and occupational outcomes.^50^^,^^51^, ^52^, ^53^, ^54^ Nurses who were exposed to infected patients or infected colleagues reported increased sleep disturbance (47% compared to 37% of non-frontline nurses), and higher turnover intention.^50^^,^^53^^,^^54^ Some of the factors identified to have increased psychological distress during the COVID-19 pandemic are long working hours, high workloads, fear of infection, and insufficient protective measures (i.e., lack of personal protection equipment).^53^, ^54^, ^55^ Furthermore, a scoping review reported that maternity staff, including nurses, reported moral distress related to excluding the patient's family from consultations and from the birthing experience.^11^ Despite this, some evidence suggested that the negative aspects of the COVID-19 pandemic on HCWs were offset by pride in their jobs and increased collegial cohesion.^11^

### Workload, staff shortages, and autonomy

Workload and its impact on HCWs is one of the most widely discussed factors relating to HCW psychological distress.^19^^,^^21^^,^^23^^,^^24^^,^^48^, ^49^, ^50^^,^^53^^,^^57^, ^58^, ^59^ Staff shortages were regularly discussed alongside heavy workloads.^19^^,^^39^^,^^49^^,^^58^^,^^60^, ^61^, ^62^ Staff shortages lead to increasing workloads, long working hours, rigid night shifts, enforced overtime, and a ripple effect of other issues, including insufficient leadership support and unrealistic expectations placed on staff by the organisation. In a UK survey, only 34% of HCWs reported staffing levels were adequate for them to do their job properly.^63^ Across Europe, increasing service demands and limited resources are intensifying pressure on healthcare systems, worsening workloads and their negative impact on HCWs.^8^^,^^64^ Excessive workloads can impact on patient outcomes,^65^, ^66^, ^67^ and unmet system demands will lead to deterioration in population health as less people can access services and those who do are at greater illness.^68^

Staff shortages and increased workload are associated with work-related stress,^21^^,^^49^^,^^58^, ^59^, ^60^ burnout^19^^,^^23^^,^^24^^,^^53^^,^^61^ and moral distress^39^ and affects satisfaction,^58^^,^^62^ disillusionment with work,^58^ motivation,^61^ and staff turnover.^50^ Burnout associated with workload was not evenly reported across professions, with junior physicians experiencing this at higher levels than more senior physicians and permanent staff more likely to experience this than temporary staff.^53^ Workload is often measured in research as staff-to-patient ratio^65^ or volume of patients seen.^69^ Workload is a multifaceted concept considering demands, cognitive load, complexity, and expertise (e.g.,^70^). While workload measures for healthcare settings have been developed (e.g.,^70^), measures capturing multiple levels of workload measure (i.e., unit-level, individual-level) do not appear to be regularly reported (e.g.,^69^). We found that workload was not often defined within the reviews found from our searches. Of those which did elaborate HCW workloads included patient load and work intensity.^53^

Karasek and Theorell's demand-control theory of job stress^45^ indicates that mismatches between workload and job control can lead to emotional exhaustion at work. Autonomy and control were key factors impacting HCWs.^19^^,^^39^^,^^56^^,^^57^^,^^59^^,^^71^, ^72^, ^73^ Autonomy from supervisors^72^ and involvement in decision-making that affects work,^39^^,^^56^ improved staff retention,^56^ job satisfaction^72^ and wellbeing,^48^ and reduced moral distress and burnout.^72^ Evidence was mixed in this area as some reviews reported no evidence of perceived control and outcomes (job satisfaction,^73^ depression^71^). Autonomy within the role could help to alleviate some of the negative impacts of high workloads if staff feel they have greater control over how they work and prioritisation.^45^ Nurses reported that staff shortages impacted on the amount of time spent with patients and families^39^ and negatively impacted feeling like they were acting according to their values.^19^ Value conflicts were reported to impact psychological distress, particularly when organisational constraints impacted care duties.^11^^,^^19^ Concerns were raised about the impacts staff shortages have on quality of patient care,^39^^,^^60^ which can increase moral distress.^39^

While most reviews did not detail the mechanisms by which workload and staff shortages impact psychological distress, likely contributors include moral injury and inability to meet professional or organisational demands. While legislation has reduced working hours for some HCWs, like doctors, in recent years,^74^ this alone may not mitigate against psychological distress as workload has not reduced instead increasing the intensity of work in some settings^75^ and overworking is still an issue due to staff shortages.^76^ Another concern connected with staff shortages is the safety of HCWs, both in exposure to violence in their roles and suicide risk. As testament to the gravity of these concerns, a recent lawsuit has been brought against ministers in France in response to HCW suicides associated with workplace and organisational factors.^77^

### Management, leadership, and organisational culture

Organisational culture and work environment impacted psychological distress and occupational outcomes.^19^^,^^23^^,^^49^, ^50^, ^56^^,^^59^^,^^60^^,^^62^^,^^71^^,^^78^^,^^79^ Organisational culture and work environment includes setting clear goals within the team and commitment of organisation members to achieving these; supportive work atmosphere, and cohesion within teams; mutual trust between colleagues; and positive connections within the organisation. Healthcare teamwork quality is associated with patient care quality and safety; poor teamwork is associated with higher levels of patient complications and increased mortality.^80^ Organisational culture more widely has also been linked with patient outcomes, with organisational culture seen as a “culprit” and “remedy” to systemic failing of healthcare systems.^81^ Co-worker relationships had a strong association with HCWs' psychological and occupational outcomes.^11^^,^^48^^,^^49^^,^^56^^,^^57^ Feeling supported was widely discussed across reviews as important for HCWs.^49^^,^^52^^,^^54^^,^^59^ This included teamwork and support from colleagues,^19^^,^^61^^,^^62^^,^^71^^,^^73^ organisational support,^46^^,^^50^^,^^54^^,^^56^^,^^62^ and the role of management and leadership.^19^^,^^49^, ^50^^,^^56^, ^57^, ^58^, ^59^, ^60^, ^61^, ^62^^,^^71^^,^^82^ Psychological distress outcomes associated with lack of support included burnout,^19^^,^^71^ depression,^71^ job dissatisfaction,^62^^,^^73^ stress,^49^^,^^58^ and morale^59^; in addition to intention to leave and staff turnover.^50^^,^^56^ Management and leadership are core components of organisational support.^19^^,^^49^, ^50^^,^^56^, ^57^, ^58^, ^59^, ^60^, ^61^, ^62^^,^^71^ Strong supervision and management support could be protective to psychological distress. Unresponsive or inexperienced managers and lack of leadership exacerbate stress, low morale, and low satisfaction.^49^^,^^58^^,^^59^ Management positions themselves come with additional stress^59^ and resilient management can foster a positive work environment to promote resilience in others.^82^ Receiving value and recognition increases motivation in HCWs,^59^^,^^61^ reduces staff turnover,^57^ increases job satisfaction,^72^ and reduces burnout.^54^^,^^72^ Hierarchical systems, punishment and risk-aversive cultures, and setting unrealistic organisational expectations on staff can increase stress, pressures, and mistrust in HCWs.^49^^,^^59^

While some occupational risk factors identified in our review (e.g., good management and staff support) may be applicable across all occupations, there are important considerations from a healthcare perspective whereby effective operation of healthcare systems and protecting HCWs from mental disorders are crucial to protecting patient outcomes and failure to do so can lead to moral and psychological distress. Other stressors, like staff shortages and patient load, are projected to worsen over time in healthcare contexts and without addressing these we are likely to see associated rises in HCW psychological distress and adverse occupational outcomes.

### Workplace bullying and violence

Support from co-workers improved staff retention and reduced turnover and psychological distress^51^^,^^57^ while conflict and aggression from staff contributed to it.^49^^,^^51^ Bullying in the workplace (e.g. verbal aggression, withholding of information, humiliation) was highlighted as a serious risk factor across numerous reviews focussing on nurses.^51^^,^^56^^,^^83^ Bullying was linked bidirectionally to mental health,^51^ and associated with anxiety symptoms, depression, distress, burnout, fatigue, sleep disorders,^51^^,^^83^ PTSD,^83^ poor wellbeing,^56^ and increased turnover.^51^^,^^83^ Baseline symptoms of depression, anxiety, and fatigue increased bullying risk.^51^ Nursing students and newly recruited staff were found to be at increased risk, with most bullying occurring in gender-dominant workplaces and often perpetrated by same-gender individuals, senior management, or experienced nurses.^83^

Exposure to workplace violence (physical and non-physical) is another risk factor impacting the mental health of HCWs.^47^^,^^50^^,^^51^^,^^84^ Workplace violence is often patient-related, although this can also be perpetrated by patients' relatives and friends and even other HCWs.^85^ Workplace violence may be a persistent or cumulative stressor^50^; with emergency department staff facing particularly high levels of patient-related violence.^47^ Physical violence toward staff is also prevalent in psychiatric and geriatric settings.^84^ Nurses face the highest levels of offensive behaviours (e.g., sexual harassment),^50^ with verbal and physical abuse being common.^47^ Workplace violence and exposure to traumatic experiences increase burnout and can lead to increases in psychological distress and PTSD.^47^^,^^50^ Workplace violence is associated with a decline in job satisfaction and increased turnover intention.^47^^,^^50^^,^^84^

### The role of stigma

Mental health professionals often face several types of stigma, including public, internalised, structural, and stigma by association.^86^^,^^87^ One review reported HCWs perceived mental health stigma to be high among their colleagues, which lead to a reluctance to disclose their own difficulties and negatively impact help-seeking.^86^ HCWs report disclosure/confidentiality concerns and negative social judgement as a barrier to mental health-related help-seeking more highly than other population groups.^88^ Barriers to help-seeking among mental health professionals include lack of resources, the devaluation of mental health at the workplace, and a culture of ‘duty before self’ which fosters an expectation to keep working even when they are unwell.^86^ Fear of letting colleagues and patients down is reportedly one of the greatest barriers to help-seeking.^89^ A rapid systematic review of psychological distress in HCWs during infectious disease outbreaks reported that perceived social stigma, which can manifest as avoidance and maintaining social distance,^87^ toward HCWs was consistently associated with higher levels of depression, anxiety symptoms, stress, PTSD and psychological distress.^54^

### Financial factors

Earnings and salaries were mentioned across reviews as a factor relating to job satisfaction,^62^ retention and turnover intention.^57^^,^^90^ Midwives often reported insufficient earnings, and that their wages did not match their responsibilities and experience.^57^ This was echoed among nurses, who report pay not matching their level of education as a stress factor leading to high turnover and intention to leave.^90^ Low salaries, particularly paired with difficult job conditions, were reported as one of the main reasons for low job satisfaction among nurses.^62^

### Individual-level risk factors and intersectionality

Younger age, identifying as a woman, being single or childless, and lower socioeconomic status was consistently associated with increased risk of stress, burnout, psychological distress, and turnover (within or out of healthcare) among HCWs.^21^^,^^23^^,^^24^^,^^46^^,^^50^^,^^53^^,^^57^^,^^58^^,^^72^ Higher education was protective in some contexts,^72^ but among migrant nurses it was linked to greater willingness to leave their profession.^57^ One review found that being married with children increased stress during COVID-19,^53^ and HCWs in low-resource settings were more likely to leave their roles during the pandemic.^50^ Psychosocial and health-related risk factors include low social support, poor sense of purpose, maladaptive coping (e.g., escape-avoidance and self-blame), and unhealthy behaviours such as poor sleep, inactivity, smoking, alcohol misuse, and poor diet.^11^^,^^51^^,^^54^ These lifestyle factors may also act as coping responses to psychological distress thus making the direction of association unclear. Additionally, psychological factors such as burnout, anxiety symptoms, depression, and stress were found to be key predictors of turnover intention.^50^

While various of these individual-level risk factors are not unique to healthcare and may be present across occupational settings, their intersection with the healthcare context, particularly when combined with systemic inequalities, can lead some HCWs to be more vulnerable. For example, nurses and midwives constitute approximately 61.3% of European HCWs, with most being women,^91^ and the proportion of internationally trained nurses varies across European states, from 0.2% in Estonia to 49.1% in Ireland.^8^^,^^92^ Official European statistics on HCWs do not report on ethnicity^92^; but in the UK 24.2% of all HCWs are from ethnic minorities.^93^

Nurses and HCWs from ethnic minorities are at increased risk of reporting harassment and bullying and thus, to be susceptible to psychological distress and negative occupational outcomes. Racism, discrimination, and stigma were commonly reported by migrant nurses. Furthermore, HCWs from ethnic minority backgrounds and immigrants face additional risks, such as unfavourable working conditions, limited opportunities for career development, and devaluation of their skills and qualifications (Panel 2). These inequalities were exacerbated during the COVID-19 pandemic, when they were more likely to be assigned to roles with higher exposure to infected patients, often with inadequate access to personal protective equipment. These groups are essential to the functioning of healthcare in Europe but experience interpersonal level risk factors at higher levels than their White or domestically trained colleagues. While some research has focused on migrant workers, other marginalised groups remained under-researched. The COVID pandemic highlighted poorer outcomes in ethnic minority HCWs,^97^ highlighting the need for further investigation into the longer-term impacts and underlying systemic inequalities.

Panel 2 **Ethnic minority and migrant HCWs experience discrimination and workplace aggression.**

In a review of the experience of ethnic minority HCWs in the UK during the COVID-19 pandemic, HCWs from ethnic minority groups were identified as being at higher risk of poor mental health outcomes, including anxiety symptoms, depression and PTSD, compared to the White ethnic group.^55^ Contributing factors included unequal distribution of Personal Protective Equipment (PPE), higher levels of discrimination in the form of impeded career advancement, direct harassment, and devaluation of professional skills, and higher contact with COVID-19 patients.^55^ Furthermore, migrant workers report stress from complex migration and nursing board registration processes, especially when placed in roles below their qualifications and skill level.^57^^,^^94^ Migrant workers are willing to stay in the host country if visa conditions are favourable but challenges securing long-term residency and family immigration often prompt relocation.^57^

Discrimination and racism were common experiences reported by migrant or internationally qualified nurses.^94^^,^^95^ One review focussing on geriatric care settings^94^ reported that migrant nurses of colour experienced overt racism, including racist remarks regarding the colour of the skin and their nationalities, in addition to refusal of care from patients. Workplace discrimination was also commonly experienced by migrant nurses,^94^^,^^95^ with assumptions that migrant HCWs were incompetent.^96^ Migrant nurses often experienced less favourable working conditions (e.g., poor timing of shifts) and had fewer opportunities for career progression than local colleagues.^94^ Social isolation and exclusion in the workplace, not feeling trusted by colleagues, communication difficulties, such as difficulties understanding colloquial terms, unfair conclusions about work ethic, and undermining of skills or qualifications were other common experiences reported.^94^^,^^95^ Manager and colleague support were shown to reduce turnover among migrant nurses,^57^ many of whom are from an ethnic minority background; however, if they experience bullying, this support becomes ineffective.

## Interventions to prevent or address psychological distress and occupational outcomes in HCWs

While some individual level approaches, such as mindfulness and resilience training, are theoretically promising and show short-term benefits, the overall quality of evidence remains limited. An umbrella review on mindfulness training for HCWs suggested improvements in psychological distress.^98^ However, a meta-analysis of seven studies reported a medium effect size for stress reduction (r = 0.34), although publication bias was suspected,^99^ and a smaller meta-analysis of two studies on nurses suggesting non-significant improvements on burnout.^100^ Furthermore, two Cochrane reviews (44 and 117 randomised control trials each) found that resilience training improved resilience and reduced depression and stress but had no effect on anxiety symptoms or wellbeing.^101^ Stress-reduction interventions, whether targeting the stressor, stress experience, or both, reduced stress in the short and medium term, with the strongest effect seen for combined interventions.^102^ However, both reviews rated the certainty of evidence as low due to risk of bias and small study samples.^101^^,^^102^ Other reviews found technology-based interventions (e.g., phone calls, web applications, or video platforms) reduced distress during COVID-19, especially with frequent contact from a mental health professional was provided.^103^ Art and music therapy showed moderate reductions of psychological distress for palliative care staff,^104^ and grief-focused interventions showed some benefits, especially for multi-day formats, reflective, and interactive formats.^78^ Despite evidence indicating generally positive effects of individual-level interventions, the findings vary in quality and strength. Given the role workplace stressors play on HCW psychological distress, the onus should not fall solely on individual resilience; interventions should be implemented alongside broader organisational strategies to support HCWs.^105^

Organisation-level interventions target system-level change, addressing root causes like workload, poor leadership, and lack of autonomy, and thus represent key leverage points where shifts can produce large-scale, lasting improvements in HCWs' mental health.^106^ UK evidence suggests that organisational interventions can reduce burnout and improve retention, especially when they address demands and resources at multiple levels and are staff-led, context-specific, and sustainable.^105^ A meta-analysis including 15 RCTs and 37 cohort studies found that organisational interventions (e.g., changes to work hours and how work is organised/carried out) were more effective in reducing burnout levels (12% reduction, 95% CI 6–17%) than individual-focused interventions (6% reduction, 95% CI −2 to 14%).^107^ Moreover, a UK review highlighted that structural changes like self-rostering improved nurses' work-life balance and job satisfaction more effectively than individual-focused strategies.^108^ Other reviews reported limited but positive evidence that HR practices, teamwork, and job design improved HCW psychological distress,^73^ while workplace health promotion interventions (stress, psychosocial, leadership, physiotherapy, and ergonomics training) showed promise but lacked strong evidence for reducing psychological distress.^109^ However, the quality of this evidence is currently insufficient, and better, rigorously designed organisation-level interventions are needed, especially for violence prevention. There is a lack of evidence assessing the impacts of healthcare policies on HCW mental disorders and retention. Further research is needed to understand the longer-term benefits and sustainability of interventions and preventative programmes on mental disorders in HCWs.

## Limits to the evidence base of mental disorders in European HCWs

Our approach to gathering evidence for this review included an umbrella review to sourcing and synthesising evidence on mental disorders in HCWs, and the role these may play in the healthcare workforce crisis. The review highlighted an association between work-related stress and psychological distress; suggesting this is elevated in high-pressure clinical environments. Quality appraisal of the included reviews showed the quality of reviews varied (see Supplementary Table S2). Reviews were predominantly based on cross-sectional data. This indicated a need for more high-quality, longitudinal research in this area.

The evidence on work-related psychological distress reflects interactions between risk and protective factors at individual, workplace, and broader environmental levels^44^; with structural organisation-based factors central to adverse outcomes. However, alongside the insights provided by the available evidence, this umbrella review also highlighted limitations to the current evidence base that restricts our understanding of mental disorders in HCWs, particularly with regards to the reporting and measurement of mental disorders, representation of varied HCW occupations, studies comparing HCWs with other populations, and coverage across European countries.

**Fig. 2:**
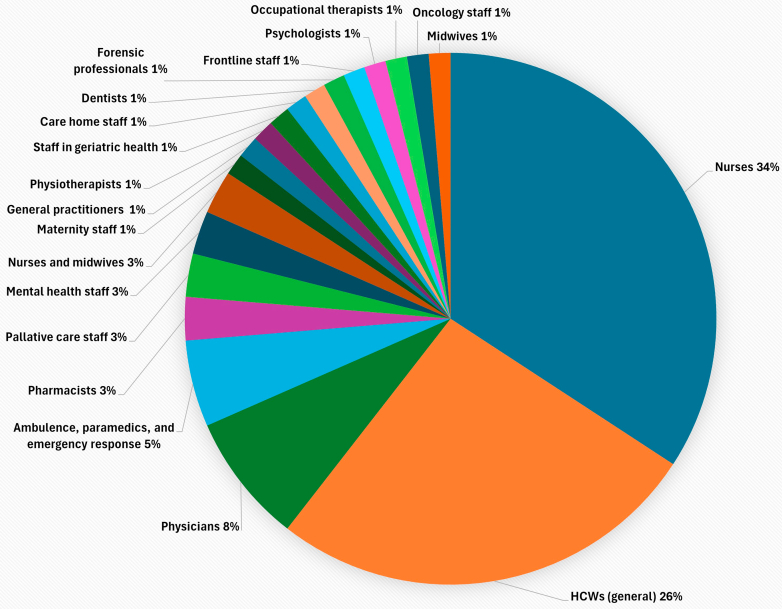
**Distribution of HCW population across included reviews**.

Most evidence was based on HCWs' self-reports; and while this indicated a high burden of psychological distress, burnout was largely the focus of most research. Mental disorders were rarely reported. Nurses were the population most studied; to a greater extent than general (i.e., non-specific) HCW populations among the reviews we report on (see Fig. 2). While nurses have been identified in comparative research as a particularly vulnerable group, perhaps justifying specific focus of much of the research in this area on them, there is a need for more research across different occupation groups to fully understand the extent of the healthcare workforce crisis and any unique stressors across groups. Support staff and lower paid HCWs likely face additional challenges to those discussed in this review but they were not identified from our search. Additionally, previous research has suggested that mental health risks in nursing may predate professional exposure,^110^ with some drawn to the profession due to lived experience of mental disorders.^111^ Research should therefore also examine this pre-existing vulnerability.

This review aimed to inform best practices in Europe, but region-specific findings were often unclear in reviews that combined evidence from European and non-European populations. 30.2% of included reviews from our searches were European only and the rest included data from integrating both European and non-European countries. Therefore, reported findings are likely to be influenced by data from non-European countries, such as the USA, Canada and Australia, as these were represented in a large proportion of included reviews. While this broader literature addresses similar challenges and offers useful insights, some caution should be taken in the generalisability of the review findings to specific European healthcare settings. There are very few findings including papers from Eastern European nations, with findings dominated by results from a large sub-set of Western Europe states (e.g., UK, Germany, France, Italy). These countries have their own challenges in this area, for example, one in five physicians in Hungary and Estonia are aged 65 years or over^112^ and Romania tackling trends of high migration of HCWs.^113^ We recognise that limiting inclusion to English-language publications excluded relevant studies in local European languages would impact ability to explore this. Variations in healthcare systems across Europe likely have an impact on staff experiences, outcomes, and policy implementation, but available data did not allow exploration of these differences (Panel 3).

Panel 3 **Summary of recommendations for policy and practice from identified reviews as well as overarching recommendations developed through synthesis of findings.**

**Workplace safety & support**

• Promote good leadership and ensure supportive and fair management practices, including efforts to prevent workplace violence, bullying, and discrimination
• Enhance job security through permanent contracts and legal support in workplace investigations
• Improve working conditions by reducing shift work burden, ensuring adequate staffing and fair pay, and providing flexible scheduling
• Provide safe transport, personal protective equipment, and better infrastructure for HCWs

**Interventions**

• Implement multi-level mental health initiatives grounded in evidence, validated by research, and piloted with staff to ensure relevance and uptake
• Ensure easy and timely access to psychological resources, including support services
• Integrate mental health education into healthcare training curricula
• Reduce stigma and promote help-seeking through organisational campaigns, exposure to staff who have benefitted from mental health support, and leadership engagement

**Organisational & policy changes**

• Strengthen leadership and managerial support through structured training, supportive environments and clear processes for whistleblowing and conflict resolution, supported by accountability and monitoring
• Improve organisational trust and workplace culture to support retention
• Ensure sustainable implementation of leadership and mental health programmes at all levels
• Monitor the mental health and job satisfaction of HCWs to inform organisational and policy reforms
• Actively engage HCWs in co-producing mental health policies and workplace changes
• Recognise and address challenges faced by specific vulnerable groups, such as migrant HCWs
• Support long-term investment in HCWs through national polices that prioritise recruitment, training and retention, and avoid unethical recruitment practices (e.g., from low-income countries)
• Implement evidence-based mental health programmes at national and local levels
• Introduce staffing mandates, legal protections against overwork, and fair pay policies to promote sustainable and healthy work environments

**Future research needs**

• Conduct high-quality research on the prevalence of mental disorders and occupational outcomes among HCWs in Europe, using objective, standardised measures and rigorous sampling to improve generalisability
• Assess the effectiveness of structural and individual-level interventions through robust study designs
• Investigate the long-term impact of sustainability for mental health programmes in healthcare settings
• Improve methodological consistency in the field, including standardisation of outcome measures
• Examine the effects of healthcare polices on workforce mental health, job-satisfaction, and retention

## Conclusion

This review provides evidence for a substantial burden of psychological distress among HCWs in Europe, with burnout and moral distress emerging as areas of concern. The evidence suggests that structural factors, such as staff shortages and poor leadership, are important contributors to increased psychological distress and occupational outcomes. However, current evidence is limited by poor representation across Eastern European regions, lack of evidence from non-clinical and marginalised staff groups, and inconsistent use of validated measures for psychological distress outcomes such as burnout. High-quality longitudinal research is needed to improve measurement of mental disorders, assess long-term impacts, and compare HCWs to other occupations and to the general population. Interventions must go beyond individual-level interventions and target systemic and organisational reform, supported by national policy, regulation, and investment.

## Contributors

PAM & SL contributed to conceptualisation, methodology, formal analysis, validation, writing-original draft, and writing-review & editing. BD contributed to formal analysis, validation, writing-original draft and writing-review & editing. BC contributed to writing-original draft and writing-review & editing. AS contributed to validation and writing-review & editing. DL, RB, RR, & SW contributed to conceptualisation and writing-review & editing. AM & CR contributed to writing-review & editing. SS & NG contributed to conceptualisation, project administration, supervision, and writing-review & editing.

## Declaration of interests

NG is president of the Society of Occupational Medicine and is managing director of March on Stress. SW is a mental health advisor on the NHS England Board. No other authors declare competing interests.
