# Supplementary material for: Futureproofing the healthcare workforce in Europe: understanding and addressing psychological distress and occupational outcomes
Source: Lancet Reg Health Eur. 2025 Oct 6;57:101463. doi: 10.1016/j.lanepe.2025.101463 (PMC12541638; doi:10.1016/j.lanepe.2025.101463)
Supplement: Supplementary Materials [file mmc1.pdf]

## Supplementary material

Futureproofing the healthcare workforce in Europe: Understanding and addressing psychological distress and occupational outcomes

## Methods

We conducted an umbrella review to collect, and review published meta-analyses, systematic reviews, meta-syntheses and other forms of evidence synthesis examining mental disorders and related occupational outcomes in HCWs in Europe. Our review was used to develop a conceptual framework that identifies key risk and protective factors, mapping determinants of HCWs mental disorders and their impact on occupational outcomes. This umbrella review was conducted following the Preferred Reporting Items for Systematic Reviews and Meta-Analyses (PRISMA) guidelines. The protocol was registered in PROSPERO [CRD42025607719].

### Search strategy.

A systematic search of the literature including of titles, abstracts, and full text was conducted in five electronic databases: Cochrane Library, Medline, PsycInfo, Embase, AMED (Allied and Complementary Medicine Database), and CINAHL (Cumulative Index to Nursing and Allied Health Literature. To ensure relevance to contemporary healthcare contexts, the search was restricted to studies published between 2010-2024. The search strategy was designed to capture studies examining the mental disorders and occupational outcomes of HCWs in Europe using, MeSH terms and keywords related to healthcare personnel (e.g., "health workforce", "nurse", "doctor", "physician", "medical staff"), mental disorders and occupational outcomes (e.g., "mental disorders", "depression", "anxiety", "suicide", "burnout", "retention", "satisfaction") were applied. Search terms included key words relating to mental disorders and healthcare workers (Supplementary Table 1). Boolean operators were used to combine key words and search strategies adapted to specific databases.

### Selection criteria.

We included peer-reviewed-papers about HCWs which-reported findings from at least two studies based in European healthcare settings (including primary, secondary, and tertiary care). HCWs were defined broadly to include both clinical staff (e.g., physicians at any level, nurses, dentists) and non-clinical staff (e.g., administrative, human resources, and managerial personnel). European countries were defined according to the WHO regional classification. Studies that included data from non-European regions were eligible if findings

from Europe were also included. Eligible study designs included systematic reviews, meta-analyses, scoping and rapid reviews, and reports that reviewed empirical studies.

We excluded publications not available in English, studies focused exclusively on students (e.g., medical or nursing students) or social care workers, and non-empirical formats such as opinion pieces, commentaries, letters to the editor, conference abstracts, policy briefs, and book chapters.

### Study selection.

Search results were managed using Rayyan (Professional version). After removing duplicates, three reviewers (PAM, SL, BD) independently screened title and abstract and full texts (PAM and SL 40% and BD (20%). 10% of all studies were further assessed by a second reviewer. Studies that did not clearly meet inclusion criteria during screening  were discussed and assessed by consensus.

### Data extraction

Data from included studies were independently extracted by four reviewers (PAM, SL, BD, AS) using standardized datasheets (Microsoft Excel). Each reviewer assessed a subset of the studies (PAM and SL 30% each, BD and AS 20% each). Extracted data included: author, year, country, review type, number of studies included, study population, exposure/intervention and control (where relevant), primary and secondary outcomes, outcome measurement tools, number of participants and participant characteristics (for meta-analysis only), and key findings. For reviews including both European and non-European studies, European only information was extracted separately when provided.

### Assessment of quality.

Study quality was assessed using the Joanna Briggs' Institute Critical Appraisal Tool for Systematic Reviews. Each of the eleven criteria was scored as 1 ("yes") or 0 ("no"/"maybe"). The quality of studies was categorized as low (0-5), medium (6-8) or high (9-11) quality. See Supplementary Table 2 for results of assessment of quality

**Supplementary Table 1. Terms and keywords used for database searches**

| | |
|---|---|
| **Databases** | Embase, Cochrane library, Medline, CINAHL, PsycINFO, AMED |
| **Population** | **MeSH terms** |
| | Health personnel OR Health occupations OR Allied health personnel OR Health workforce |
| | **Keywords** |
| | nurs*<br>doctor*<br>physician*<br>(“medical staff”)<br>(“support worker*”)<br>(dentist* OR “dental staff”)<br>(“health care work*” OR “health* work” OR HCW)<br>(“health* personnel” OR “health care personnel”)<br>(“health* staff” OR “health care staff”)<br>(“health* assistant*” OR “health care assistant*”)<br>(“health* professional*” OR “health care professional*”)<br>(“health* employ*” OR “health care employ*”)<br>(“health* practitioner*” OR “health care practitioner*”) |
| **Outcome** | **MeSH terms** |
| | Mental Health OR Mental Disorders OR “Burnout, Professional” OR Depression OR Anxiety disorders OR “Stress Disorders, Post-Traumatic” OR “Stress, Psychological” OR Suicide (including attempt) OR Substance-related disorders OR alcohol abuse OR alcohol consumption |
| | **Keywords** |
| | anxi*<br>help*<br>stigma<br>distress<br>absence<br>depress*<br>alcohol*<br>pressure<br>retention<br>addiction*<br>insomnia<br>psychosis<br>substance*<br>psychiatric – consider specifying “psychiatric illness”<br>satisfaction<br>psychol*<br>(“mental health”)<br>(“mental disorder*”)<br>(“quality of life” OR QoL)<br>(“burn out” OR “burn-out” OR “burnout”)<br>(“well being” OR “well-being” OR “wellbeing”)<br>(“suicide*” OR “suicid* thoughts” OR “suicid* attempt*”)<br>(“mental* symptoms“ OR “mental health symptoms”)<br>(*stress OR “psychological *stress”)<br>(“post traumatic stress disorder“ OR “post?traumatic stress disorder“ OR PTSD) |
| **Limits** | **Time frame** |
| | 2010-current |
| | **Study type** |
| | Review<br>Systematic review<br>Meta analys?s<br>Synthesis<br>Summar* |
| | **Region** |
| | Europe<br>European Union<br>Developed countries<br>High income countries<br>OECD countries |

**Supplementary Table 2. Summary of findings from umbrella review on mental health of healthcare workforce in Europe**

| ID | Author, year | Title | Countries | Study design | N studies included | Population. Setting (if available) | Outcome. Measurement tools used (if available) | Key findings | Quality classification[a] |
|---|---|---|---|---|---|---|---|---|---|
| **1** | Villamin et al., 2024 | Retention and turnover among migrant nurses: A scoping review | Europe and other | Scoping review | 16 | Migrant nurses. Any clinical setting | Retention, turnover and turnover intentions: No specific measure | Staff retention, turnover, and turnover intentions among migrant nurses in different countries. Factors influencing staff retention, turnover, and turnover intention included a variety of personal, organisational, financial, political and environmental characteristics. | Moderate |
| **2** | Gupta et al., 2024 | Mental health stigma and its relationship with mental health professionals - A narrative review and practice implications | Europe and other | Narrative review | 387 | Mental health professionals. Mental healthcare | Mental health-related stigma: No specific measure | Stigma towards mental health was shaped by organisational culture, leadership, and training. Many mental health professionals who experience mental health conditions experience stigma, which impact decisions to access care. Stigma may include fear of disapproval from colleagues and anticipated negative impacts to career growth opportunities. Not accessing care can lead to an over-reliance on self-treatment, low perceived peer support, and an increased risk of self-harm | Low |
| **3** | Schmitt et al., 2021 | Effects of the Covid-19 pandemic on maternity staff in 2020 - a scoping review | Europe and other | Scoping review | 54 | Maternity staff. Maternity care | Impacts caused by the COVID-19 pandemic: No specific measure | Organisational and structural challenges created by the COVID-19 pandemic (e.g. providing normal care despite changing guidelines and protocols, or increased workload due to higher number of staff off sick) pushed many maternity staff outside of their "comfort zone," which impacted workforce mental health and service delivery. | Moderate |
| **5** | Braquehais et al., 2015 | Service provision to physicians with mental health and addiction problems | Europe and other | Narrative review | Not reported | Physicians. Any clinical setting | Nature and development of programs that specifically treat sick doctors: No specific measure | Physicians often don't ask for help when experiencing substance use disorders and other mental illnesses. Data exploring Physician Health Programmes in Europe and other | Low |

| | | | | | | | | | |
|---|---|---|---|---|---|---|---|---|---|
| | | | | | | | | regions showed mixed, but generally good evidence on effectiveness in supporting healthcare workers' mental health and promoting safe practice. | |
| **6** | Bronkhorst et al., 2015 | Organizational climate and employee mental health outcomes: A systematic review of studies in health care organizations | Europe and other | Systematic review | 21 | HCWs. Any job role and clinical setting | Mental health: No specific measure | With mixed results, strong organisational climate (e.g. experiences with leadership, group relations, and communication) was generally associated with reduced psychological distress and improved mental health among healthcare workers. Good leadership and strong group relations appeared important, though most studies exploring communication found no association with mental health outcomes. | Low |
| **7** | Hassell et al., 2011 | Workload in community pharmacies in the UK and its impact on patient safety and pharmacists' well-being: A review of the evidence | Europe: UK | Literature review | 15 | Pharmacists. Community pharmacy | Impact of increasing pharmacist workloads on outcomes such as job satisfaction: No specific measure | While there was limited evidence on impact of highlight workload and work-related stress on mental health outcomes, there was more evidence on occupational outcomes. Higher workload and work-related stress were associated with increased intention to leave job and lower job satisfaction. | Moderate |
| **8** | Nichols et al., 2010 | The experiences of internationally recruited nurses in the UK (1995-2007): An integrative review | Europe: UK | Integrative review | 30 | Migrant nurses. All clinical settings | Experience of living and working in the NHS among non-UK born/trained nurses: No specific measure | Five themes were developed in the review, which looked at nurses motivations for migrating to the UK, their experiences of adapting to working in the UK, their experiences with so-called "first world healthcare" compared to in their home country, feelings of being devalued or becoming deskilled in their new role, and experiences of racial discrimination. Experiences in these vectors impacted their job satisfaction, intention to stay in the UK, and their mental health and wellbeing. | Moderate |

| | | | | | | | | | |
|---|---|---|---|---|---|---|---|---|---|
| **10** | Di Mario et al., 2023 | The use of yoga and mindfulness-based interventions to reduce stress and burnout in healthcare workers: An umbrella review | Europe and other | Umbrella review | 12 | HCWs. Any job role and clinical setting | Burnout: MBI (Maslach Burnout Inventory)<br>Stress, anxiety, depression, burnout and sleep disorders: No specific measure | Systematic reviews found that yoga interventions can reduce musculoskeletal and psychological problems and stress among HCWs. Reviews also found that mindfulness-based interventions can effectively reduce burnout, and other outcomes such as physical and mental health, stress, and job performance. Interventions that incorporate both yoga and mindfulness-based elements likely produce similar benefits. | Moderate |
| **11** | Park, 2021 | Meta-analysis of factors associated with occupational therapist burnout | Europe and other | Meta-analysis | 17 | Occupational therapists. Any clinical setting | Burnout: MBI, OBI (Oldenburg Burnout Inventory) and BM (Burnout Measure) | The variables with significant positive associations with increased burnout were job challenges (unclear), marital status (being unmarried), patient age (being older), position (being a supervisor), turnover intention (more likely to leave), working type (unsure what this includes), work addiction (more addicted), work field (working in a large hospital), and working hours (working more hours). Among these, the effect size of marital status, work field, and work hours was small, while those of job challenges, patient age, position, turnover intention, working type, and work addiction was medium. Variables with significant negative effects on burnout were age (being younger), education (less education), engagement (less engagement), job satisfaction (less job satisfaction), personal identity (unclear), professional identity (unclear), and feeling valued (feeling less valued). All negative significant effect sizes were medium. | Moderate |
| **13** | Suleiman-Martos et al., 2020 | The effect of mindfulness training on burnout syndrome in nursing: A | Europe and other | Systematic review and meta-analysis | 17 | Nurses, including those in management. Oncology, PICU, ICU, and geriatric | Burnout: MBI, ProQoL (Professional Quality of Life), and CBI | All included studies found that mindfulness-based interventions reduced the emotional exhaustion dimension of burnout | High |

| | | | | | | | | | |
|---|---|---|---|---|---|---|---|---|---|
| | | systematic review and meta-analysis | | | | | (Copenhagen Burnout Inventory) | among HCWs, while there were mixed results regarding it's impact on the depersonalisation and personal accomplishment dimensions. Mindfulness-based interventions can be effective even if they are short, but more evidence is needed to establish long-term benefits. | |
| **15** | West et al., 2016 | Interventions to prevent and reduce physician burnout: A systematic review and meta-analysis | Europe and other | Systematic review and meta-analysis | 52 | HCWs, mostly physicians. N/A | Burnout. Maslach Burnout Inventory. | Structural interventions, including changes in duty-hours, were effective than individual approaches in reducing physician burnout, leading to significant improvements in overall burnout. | High |
| **16** | Wilson, 2016 | An exploration of bullying behaviours in nursing: A review of the literature | Europe and other | Literature review | 28 | Nurses. N/A | Bullying. N/A | Incidence of bullying highlighted as a serious issue, particularly among nursing staff. Prevalence of bullying ranged from 14.7% to 44% of staff indicating current or previous bullying behaviour. These behaviours included having opinions and views ignored, colleagues withholding information, being given unreasonable workloads. Bullying was related to hierarchical dynamics and results in significant psychological harm. | Low |
| **17** | Peters et al., 2012 | Is work stress in palliative care nurses a cause for concern? A literature review | Europe and other | Literature review | 16 | Nurses. Palliative care | Stress and burnout. Nursing Stress Scale (NSS), Nurse Stress Index (NSI), Nurses Coping with Stress Questionnaire (NCWSQ), Job Diagnostic Survey (JDS), Profile of Mood States (POMS), Job Content Questionnaire (JCQ}, Effort-Reward Imbalance Questionnaire (ERI-Q), Depression Anxiety and Stress Scale (DASS 21), Health and Safety Executive | Stress and burnout among palliative care nurses were influenced by organisational and environmental factors, including role ambiguity, high workloads and poor communications among providers. Stress was buffered by supportive work environments and strong personal coping strategies. | Low |

| | | | | | | | | | |
|---|---|---|---|---|---|---|---|---|---|
| | | | | | | | Management Standards Indicator Tool (HSE MSI), Maslach Burnout Inventory (MBI), Professional Quality of Life Scale (ProQoL), Ways of Coping Scale (WOCS) | | |
| **18** | Lombardo et al., 2024 | Burnout and Stress in Forensic Science Jobs: A Systematic Review. | Europe and other | Systematic review | 10 | Forensic professionals. Healthcare and forensic laboratory | Burnout, stress, PTSD | High burnout levels across forensic professionals with particularly high levels among women. Burnout was increased by secondary trauma, especially from handling emotionally distressing cases such as child autopsies. A lack of organizational support and resources, along with sociodemographic disparities, significantly contributed to burnout severity. | Moderate |
| **19** | Marzocchi et al., 2024 | Job demands and resources and their association with employee well-being in the European healthcare sector: a systematic review and meta-analysis of prospective research. | Europe | Systematic review and meta-analysis | 46 | HCWs. Hospitals, primary care, administrative healthcare settings | Burnout, distress, absenteeism, sleep difficulties | Job demands including threat related (e.g., workplace violence, emotional burden), hindrance related (e.g., role ambiguity, work-life conflict), and challenge related (e.g., cognitive load, patient contact), were drivers of burnout and stress. Challenge demands contributed to work related strain but not to motivation and growth. Supportive job resources like control, fairness, and peer support helped reduce strain and improve motivation. Factors influencing staff retention and turnover included workload, support, and career development. | Low |
| **20** | Venturini et al., 2024 | Prevalence of burnout among physiotherapists: a systematic review and meta-analysis. | Europe and other | Systematic review and meta-analysis | 17 | Physiotherapists. Hospitals, rehabilitation centres, and private practice | Burnout, emotional exhaustion, depersonalisation, low personal accomplishment | The pooled prevalence of overall burnout among healthcare workers was 8% (95% CI: 4–15%). Emotional exhaustion was the most common symptom (27%), followed by low personal accomplishment (25%) and depersonalisation (23%). Burnout rates were generally higher in developing countries | Moderate |

| | | | | | | | | | |
|---|---|---|---|---|---|---|---|---|---|
| | | | | | | | | compared to developed ones, although this difference did not reach statistical significance. | |
| **21** | Yang et al., 2024 | Risk factors and consequences of mental health problems in nurses: A scoping review of cohort studies. | Europe and other | Scoping review | 171 | Nurses. Hospitals, community healthcare, and clinical environments | Depression, anxiety, PTSD, burnout, sleep disorders, positive and negative mental health outcomes. | Nurses experience high levels of depression, anxiety, PTSD, burnout, and cognitive impairment, particularly in high-stress settings involving shift work, bullying, and inadequate support. These mental health challenges are strongly tied to modifiable risk factors such as job demands, lifestyle behaviours, and workplace culture, and have severe consequences including chronic physical illness, job dissatisfaction, and increased turnover. | Low |
| **22** | Alkan et al., 2024 | Organisational factors associated with healthcare workforce development, recruitment, and retention in the United Kingdom: a systematic review. | Europe | Systematic review | 10 | Nurses, therapists, paramedics, physiotherapists, dietitians. Hospitals, care homes, community nursing | Recruitment, retention and turnover | Nurse retention was negatively affected by workplace aggression (especially from colleagues), moral distress, and job stress, while flexible shift patterns and strong managerial support were linked to greater retention, including working beyond retirement | Low |
| **24** | Varela et al., 2023 | Psychological interventions for healthcare professionals during the COVID-19 pandemic: A systematic review. | Europe and other | Systematic review | 10 | HCWs. Hospitals and healthcare | Depression, anxiety, stress, PTSD | Most interventions showed positive effects on mental health outcomes Two most effective interventions had frequent contact and feedback from mental health professionals Interventions delivered by mental health professionals with frequent contact were | Moderate |
| **25** | Thompson, 2024 | Impact of COVID-19 on Ethnically Minoritised Carers in UK's Care Home Settings: a Systematic Scoping Review. | Europe | Systematic scoping review | 10 | Ethnically minoritised carers. Care and nursing homes | Anxiety, depression, PTSD, workplace discrimination | Limited access to PPE and inadequate training were associated with higher stress levels. Higher COVID-19 infection risk and mental health challenges were found among ethnically minoritised carers. | Moderate |
| **26** | Long et al., 2023 | The prevalence of professional burnout among dentists: a systematic review and meta-analysis. | Europe and other | Systematic review and meta-analysis | 15 | Dentists. Dental practice | Burnout | The overall prevalence of professional burnout among dentists was 13% (95% CI: 6-23%) Geographical differences: highest prevalence in Europe | Low |

| | | | | | | | | | |
|---|---|---|---|---|---|---|---|---|---|
| | | | | | | | | (17%), followed by Asia (12%), and lowest in the Americas (8%). The burnout prevalence in cross-sectional surveys (11%) was significantly lower than in longitudinal studies (20%). The overall burnout prevalence in the last decade (after 2010) was significantly lower (11%) than a decade ago (before 2010) (15%). | |
| **27** | Ribero Miranda et al., 2024 | Moral Distress of Nurses Working in Paediatric Healthcare Settings. | Europe and other | Scoping review | 54 | Nurses, pediatrics. Home care, hospital, PICU, NICU, paediatric oncology | Moral distress | Heightened moral distress in paediatric care arises from fast-paced, ethically complex environments, especially when treating critically ill children. Key contributors include providing disproportionate care perceived as futile, lack of professional autonomy, communication challenges around death and prognosis, and limited resources that hinder quality care. These issues reduce job satisfaction, lower care quality, and contribute to staff turnover. Moral distress is subjective and can lead either to resilience, fostering growth and advocacy, or to moral residue, resulting in unresolved distress, disengagement, and potential psychological harm. | Moderate |
| **29** | Sheehy et al., 2024 | The reported experiences of internationally qualified nurses in aged care: A scoping review. | Europe and other | Scoping review | 14 | Migrant nurses. Residential aged care, community elderly care | Stress | 1. Stress of Migration and Transition<br>Internationally qualified nurses (IQNs) faced high stress due to complex migration and nursing registration procedures. They experienced simultaneous physical, social, and professional transitions, leading to decreased job satisfaction. Many worked in roles beneath their qualification level, compounding this stress.<br>2. Communication, Racism, and Discrimination<br>Language barriers and difficulties understanding local | Low |

| | | | | | | | | | |
|---|---|---|---|---|---|---|---|---|---|
| | | | | | | | | expressions affected communication. IQNs reported overt racism from patients (often rationalised) and colleagues, alongside discrimination in work conditions and limited career progression opportunities.<br>3. Aged Care-Specific Challenges<br>IQNs were often unfamiliar with residential aged care settings and culturally different approaches to elder care, especially dementia and palliative care. Aged care was perceived as low-status ("bottom care"), contributing to fears of de-skilling and loss of professional identity, especially for those from acute care backgrounds. | |
| **31** | Shen et al., 2022 | The global prevalence of burnout among general practitioners: a systematic review and meta-analysis. | Europe and other | Systematic review and meta-analysis | 16 | General practitioners. Primary care | Burnout | Pooled prevalence rates<br>High emotional exhaustion: 37% (95% CI: 0.30-0.44)<br>High depersonalization: 28% (95% CI: 0.21-0.34)<br>Low personal accomplishment: 26% (95% CI: 0.21-0.32)<br>Higher rates found in earlier studies (2001-2009) compared to later studies (2010-2020)<br>European studies showed higher depersonalization rates than other regions | Low |
| **32** | Lanada & Culligan, 2024 | The experiences of internationally educated nurses who joined the nursing workforce in England. | England | Integrative review | 9 | Internationally educated nurses. NHS hospitals | Pastoral/Cultural Integration | Expectations:<br>Internationally educated nurses (IENs) often faced a mismatch between their expectations and reality in England. Many experienced nurses were employed as healthcare assistants, leading to feelings of de-skilling and loss of professional status.<br>Challenges:<br>They encountered cultural differences in care practices, particularly around elder care and family involvement, as well as communication difficulties | Low |

| | | | | | | | | | |
|---|---|---|---|---|---|---|---|---|---|
| | | | | | | | | with different English accents and colloquialisms. Belongingness: IENs reported feelings of loneliness, discrimination, and humiliation, including experiences of racism and unfair treatment from patients and colleagues. They also faced exclusion from planning, development, and career progression opportunities. | |
| **33** | Carvajal et al., 2023 | A global overview of midwives' working conditions: A rapid review of literature on positive practice environment. | Europe and other | Rapid review | 70 | Midwives. Primary and secondary care | Perceptions and experiences of positive practice environments | Midwives, especially in rural and low-resource settings, faced physical risks such as violence, unsafe commutes, poor infrastructure, and lack of PPE. Many newly qualified midwives had limited job security, often relying on temporary contracts. While autonomy was valued, it was frequently restricted in physician-led hospitals, though rural and caseload roles offered greater satisfaction. Training access was important but limited by cost and inequality. High workloads, staff shortages, and exposure to trauma contributed to burnout, compounded by poor institutional support. Work-life balance was difficult to maintain, and low pay with few financial incentives increased dissatisfaction. Strong team support improved morale, but poor leadership, bullying, and blame cultures, especially for new and internationally trained midwives, undermined cohesion. | High |
| **34** | HaGani et al., 2022 | Burnout among oncologists and oncology nurses: A systematic review and meta-analysis. | Europe and other | Systematic review and meta-analysis | 34 | Oncologists and oncology nurses. Primary hospitals providing oncology care | Burnout: Maslach Burnout Inventory (MBI) | Oncology professionals face unique stressors such as patient suffering, grief, and ethical challenges. While burnout rates are similar between nurses and doctors, doctors report higher depersonalisation, possibly due to gender differences, as women—more common in | Low |

| | | | | | | | | | |
|---|---|---|---|---|---|---|---|---|---|
| | | | | | | | | nursing—tend to report lower levels. Burnout is driven by administrative burdens, limited organisational support, and the demanding nature of oncology work, though protective factors like work-life balance, resilience, and strong patient relationships (more common among nurses) can help. Regional differences exist: European oncologists report higher depersonalisation than those in the US, and Asian oncologists report lower personal accomplishment. Among nurses, Asian oncology nurses show higher depersonalisation than US counterparts, while Canadian nurses report lower personal accomplishment than those in Europe and the US. | |
| **35** | Alimoradi et al., 2023 | Estimation of moral distress among nurses: A systematic review and meta-analysis. | Europe and other | Systematic review and meta-analysis | 86 | Nurses. Any clinical setting | Moral distress: Moral Distress Scale–Revised<br>Moral Distress Scale Neonatal–Paediatrics<br>Moral Distress Scale COVID-19 Moral Distress Scale<br>Moral Distress for Healthcare Professionals<br>Moral Distress Thermometer<br>Single-Item Moral Distress Frequency<br>Nurses' Moral Distress Scale by Atashzadeh-Shoorideh<br>ICU Nurses' Moral Distress Scale<br>Moral Distress Scale for Psychiatric Nurses<br>Ethical Dilemmas in Nursing<br>Moral Distress Scale for Mental Health Services | Study indicated that nurses did not have high levels of moral distress. Pooled effect size of 2.55 (Possible range 0-10). Secondary analysis indicated that moral distress decreased after COVID-19. No significant association between workplace setting | High |

| | | | | | | | | | |
|---|---|---|---|---|---|---|---|---|---|
| **36** | Sutherland et al., 2023 | Fatigue and its impact on performance and health. | Europe and other | Literature review | Not specified | HCWs. N/A | Wellbeing and mental health | Shift work is associated with increase risk of mental health conditions, particularly depression. Shift work increases risk of sleep disorders; and COVID-19 worsened sleep quality and increased stress in HCWs. Poor sleep and stress in HCWs and their impacts on decision-making, psychological health and emotional reactivity have been heightened in the pandemic. | Low |
| **37** | Serra et al., 2023 | Workload, Job Satisfaction and Quality of Nursing Care in Italy: A Systematic Review of Native Language Articles. | Europe: Italy | Systematic review | 11 | Nurses. Hospitals and community | Job satisfaction: Family Satisfaction with Advanced Cancer Care-2 (FAMCARE-2) Maslach Burnout Inventory (MBI) McCloskey Mueller Satisfaction Scale (MMSS) Empowering Leadership Questionnaire (ELQ) Compassion Satisfaction scale (CS) Index of Work Satisfaction (IWS) Nursing Activities Score (NAS) Indice di Dipendenza Assistenziale (Dependency to Care Index) (IDA) Index of Caring Complexity (ICC) | Findings varied: one study found 76% of nurses were dissatisfied with organisational well-being and pay, while another reported 61% satisfaction with work. Job dissatisfaction was linked to intentions to leave, influenced by factors such as lack of recognition and growth opportunities, poor teamwork, heavy workload, and limited autonomy. One study highlighted significant mental health concerns, with 35% of nurses experiencing depression and very high rates of anxiety (74.8%), fatigue (89.6%), and irritability (86.1%). | Moderate |
| **38** | Almeida et al., 2023 | Emotional Management Strategies in Prehospital Nurses: A Scoping Review. | Europe | Scoping review | 4 | Nurses. Pre-hospital | Emotional regulation | Emotional management among healthcare professionals involves both individual and collective strategies, with a strong emphasis on personal responsibility through self-care, mental preparation, and emotional distancing. Collective approaches include peer support, teamwork, and institutional backing. High-stress situations—such as paediatric cases, | Moderate |

| | | | | | | | | | |
|---|---|---|---|---|---|---|---|---|---|
| | | | | | | | | suicides, traffic accidents, and personal risk—often trigger these strategies. Nurses commonly adopt a professional role to suppress emotions and focus on patient care. Emotional management can be formal or informal, including debriefing, training, team support, physical activity, humour, acceptance, and maintaining a positive outlook, with strategies influenced by experience and context. | |
| **39** | Lipman et al., 2021 | Staff wellbeing: a matter for quality indicators or a concern in its own right? | Europe: UK | Systematic review | 13 | Ambulance, paramedics, and emergency response staff. Ambulance/emergency response | Stress, dissatisfaction, burnout, PTSD, exhaustion, demoralisation, substance misuse, suicide, low mood | Ambulance staff face high stress, poor mental health, and the highest sickness absence rates in the NHS, driven by excessive demands, poor leadership, low pay, and lack of support. These issues contribute to high turnover, poor wellbeing, and reduced patient care quality. Mental health problems like anxiety, burnout, and PTSD are widespread, with limited access to coping mechanisms. Organisational culture, outdated practices, and restrictive performance targets (AQIs) worsen the situation. Staff wellbeing, retention, and sickness are closely linked, highlighting an urgent need for systemic reform. | Low |
| **40** | Alshawwa et al., 2023 | Exploring the impact of the COVID-19 pandemic on mental health organisations in England. | Europe: UK | Literature review | 44 | Mental health staff. Mental health organisations | Depression, anxiety, PTSD, self-harm | Four key themes emerged: organisational structure, practice approaches, leadership, and staff support. Staff support was the most prominent theme. Academic literature highlighted significant physical and mental health impacts on healthcare staff, especially nurses, including anxiety, depression, PTSD, and self-harm. Non-academic sources also reported heavy impacts, citing workload and absence management challenges. Findings showed organisations | Low |

| | | | | | | | | | |
|---|---|---|---|---|---|---|---|---|---|
| | | | | | | | | were initially unprepared to meet staff support needs. While pandemic guidelines focused on individual mental health, staff stressed the need for stronger systemic and environmental support. | |
| **42** | Hill et al., 2016 | Improving the wellbeing of staff who work in palliative care settings: A systematic review of psychosocial interventions. | Europe and other | Systematic review | 10 | Palliative care staff. Palliative care settings | Psychological distress, depression, anxiety, stress, and burnout | Interventions included organisational support, stress reduction, music and art therapy, psycho-existential and sleep interventions, ranging from one-off sessions to weekly year-long programmes. Most showed no impact on psychological outcomes, though some had secondary benefits. RCTs and several group-based programmes showed no effect, while art and music therapy showed moderate improvements. Many participants had relatively good psychological wellbeing at baseline. | High |
| **43** | Kansoun et al., 2019 | Burnout in French physicians: A systematic review and meta-analysis. | Europe: France | Systematic review and meta-analysis | 37 | Physicians. Any clinical setting | Burnout. Maslach Bunout Inventory (MBI) | Across 23 studies with 9,667 physicians, burnout prevalence ranged from 28% to 73%, with a pooled estimate of 49%. Severe burnout affected 5%, high emotional exhaustion (EE) 21%, high depersonalization (DP) 29%, and low personal accomplishment (PA) 29%. Emergency physicians had higher burnout rates (57%) and more severe burnout than others. No individual-level factors were linked to burnout in meta-regression, but higher study quality and being an emergency physician were associated with more severe burnout. Junior residents showed higher DP and lower PA, while anesthesiologists had lower EE and DP rates. | High |
| **44** | Dickson et al., 2022 | UK nurses' and midwives' experiences of healthful leadership | Europe and other | Rapid realist review | 38 | Nurses and midwives, any clinical setting | Wellbeing and resilience | Leaders who care for themselves foster authenticity and resilience, encouraging staff wellbeing and | Moderate |

| | | | | | | | | | |
|---|---|---|---|---|---|---|---|---|---|
| | | practices during the COVIDâ€19 pandemic: A rapid realist review. | | | | | | boosting morale. Informed leaders who share information and use support systems help reduce staff psychological harm. Authentic, values-driven leadership promotes a positive culture, resilience, job satisfaction, and connection among staff. | |
| **45** | Chutiyami et al., 2022 | COVID-19 pandemic-related mortality, infection, symptoms, complications, comorbidities, and other aspects of physical health among healthcare workers globally: An umbrella review. | Europe and other | Umbrealla review | 13 | HCWs. Any setting | Burnout and stress. Anxiety Sensitivity Index (ASDI); Symptom Checklist-90 (SCL-90); Impact of Event Scale – Revised (IES-R); Perceived Stress Scale (PSS); Stress Overload Scale (SOS); Self-Reporting Questionnaire (SRQ). | Six reviews reported stress associated with workload/long working hours coupled with psychological distress among healthcare workers. Five of these were high quality reviews. The prevalence of stress ranged from 33% to 44.86%. Stress was higher in female HCWs, those who are married, have children, nurses, trainees, and non-physicians. Burnout associated with workload was reported to be different among physicians with different roles, with resident physicians experiencing greater burnout than attending physicians. | High |
| **47** | Xin et al., 2022 | Psychological sequelae within different populations during the COVID-19 pandemic: a rapid review of extant evidence. | Europe and other | Rapid review | 150 | HCWs. N/A | Anxiety, depression, PTSD. Depression, Anxiety, and Stress Scale (DASS-21); Generalized Anxiety Disorder-7 (GAD-7); Patient Health Questionnaire-9 (PHQ-9); Impact of Event Scale-Revised (IES-R); Perceived Stress Scale (PSS); Insomnia Severity Index (ISI); Maslach Burnout Inventory (MBI); Post-Traumatic Stress Disorder Checklist-Civilian Version (PCL-C); Fear of COVID-19 Scale (FCV-19S); Self-Reporting Questionnaire (SRQ- | Among healthcare workers, anxiety prevalence ranged from 5.7% to 61.0%, depression from 8.9% to 64.7%, and post-traumatic stress from 3.8% to 49.4%. Overall, they reported higher anxiety (33.0%) but lower traumatic stress (14.6%) compared to the general population (24.8% and 20.8%, respectively). Compared to past outbreaks like SARS and MERS, COVID-19 saw lower rates of anxiety (33% vs. up to 96%) and traumatic stress (14.6% vs. 25.5%). | Moderate |

| | | | | | | | | | |
|---|---|---|---|---|---|---|---|---|---|
| | | | | | | | 20); Kessler Psychological Distress Scale (K-6/K-10); Hospital Anxiety and Depression Scale (HADS); Warwick-Edinburgh Mental Well-being Scale (WEMWBS); Symptom Checklist-90-Revised (SCL-90-R); Copenhagen Burnout Inventory (CBI); General Health Questionnaire (GHQ-12/GHQ-28); Pittsburgh Sleep Quality Index (PSQI). | | |
| **48** | Webster et al., 2022 | Self-rostering, work-life balance and job satisfaction in UK nursing: a literature review. | Europe: UK | Literature review | 6 | Nurses. Any clinical setting | Job satisfaction | Studies supported self-rostering as beneficial for improving work-life balance and job satisfaction.<br>Job satisfaction: Studies indicated higher levels of staff empowerment and motivation, and reduced staff attrition upon implementation of self-rostering. Self-rostering improved feelings of autonomy and collective responsibility, which increased job satisfaction.<br>Work-life balance: Self-rostering improved ratings of work-life balance, as staff were able to coordinate their lives more effectively and have better control of planning their lives outside work. Self-rostering can improve the consecutive number of days worked by nurses. | Moderate |
| **49** | Nwanya et al., 2021 | The importance of understanding burnout: an oncology nurse perspective. | Europe and other | Literature review | 31 | Oncology nurses. N/A | Burnout. Maslach Burnout Inventory (MBI) | Burnout among oncology nurses was already high before COVID-19 (ranging from 3–65%) and likely worsened during the pandemic. One review found emotional exhaustion rates between 3–38% across 27 studies, highlighting variation by setting. Most studies are small, | Moderate |

| | | | | | | | | | |
|---|---|---|---|---|---|---|---|---|---|
| | | | | | | | | cross-sectional, and methodologically weak. Burnout is largely driven by workplace factors, particularly those that conflict with nurses' values. Six key contributors include work overload, lack of control, lack of reward, poor community, value conflict, and unfairness. Despite this, interventions often target individual coping rather than systemic issues, and most are small-scale or lack strong evidence, though some mindfulness-based approaches show promise. | |
| **50** | Russo et al., 2021 | The impact of economic recessions on health workers: a systematic review and best-fit framework synthesis of the evidence from the last 50 years. | Europe and other | Systematic review | 57 | HCWs. Any | Burnout and depression | In Europe, clinical burnout, migration, and multiple jobholding were common reactions among healthcare workers, while in low-income settings, loss of motivation, absenteeism, and system appropriation were more frequently reported. Evidence from Greece, Ireland, Italy, and Spain supported predictions about healthcare worker burnout during the Great Recession. In Greece, burnout among medical researchers rose from 20% to 60%, likely due to funding uncertainty. An Italian study found major depression affected 33% of healthcare workers—twice the general population rate. Migration of healthcare workers was noted in countries like Ireland and Portugal. Overall, burnout was linked to worsening working conditions, heavier workloads, wage cuts, and limited healthcare resources. | High |
| **51** | Warren-James et al., 2022 | How do paramedics cope? A scoping review. | Europe | Scoping review | 3 | Paramedics. N/A | Coping strategies | Healthy coping strategies identified include: self-soothing, distraction, social support. Unhealthy coping strategies identified include: negative self-talk (suppression/denial), | Low |

| | | | | | | | | | |
|---|---|---|---|---|---|---|---|---|---|
| | | | | | | | | harmful activities (alcohol use). Mindful awareness (focusing in the present moment) was the most frequently reported self-soothing strategy. Social support was identified in all three studies. Professional support (GP, counsellors) and religion were both mentioned in one study, respectively. | |
| **52** | Pellegrini et al., 2022 | Secondary Trauma and Related Concepts in Psychologists: A Systematic Review. | Europe and other | Systematic review | 8 | Psychologists. Any | Secondary trauma, compassion fatigue, vicarious trauma, and secondary traumatic stress | Prevalence rates of secondary trauma among psychologists could not be determined due to a lack of studies and methodological limitations, such as varied outcome measures, unsuitable psychometric tools, and reporting of mean scores rather than clinical cutoffs. Existing evidence suggests secondary trauma generally does not cause clinically significant issues for psychologists. However, increased exposure to trauma survivors, personal trauma history, limited social support, higher PTSD caseloads, younger age, female gender, rural work settings, and less experience may raise risk. Protective factors include social support, trauma resolution, and effective coping strategies. Psychologists tend to use these strategies more than other health professionals, potentially explaining sub-threshold symptom levels. Common coping approaches included limiting trauma-specific clients, which has proven effective in managing secondary trauma. | High |
| **53** | Afolabi et al., 2018 | The effect of organisational factors in motivating healthcare employees: a systematic review. | Europe and other | Systematic review | 14 | Doctors, dentists, nurses, laboratory scientists, pharmacists and mid-level health providers. Hospitals, clinics and health centres. | Performance of healthcare workers | Poor management, lack of teamwork and a lack of support from supervisors and those in authority were observed as strong demotivating factors. Although financial incentives were considered important, they | Moderate |

| | | | | | | | | | |
|---|---|---|---|---|---|---|---|---|---|
| | | | | | | | | were not enough by themselves to motivate healthcare professionals. Staff shortages led to work overload for those still in service, which invariably led to staff burnout. | |
| **54** | Schaller et al., 2022 | Workplace health promotion interventions for nurses in Germany: a systematic review based on the RE-AIM framework. | Europe: Germany | Systematic review | 11 | Nurses. Acute medical care, inpatient long-term care, and home-based long term care | Stress, wellbeing, mental health-related quality of life. N/A | For the majority of outcomes, no statistically significant differences were seen in the effectiveness of workplace health promotion. There was some evidence that it effectively impacted perceived job stress and mental health related quality of life. | Moderate |
| **56** | Parola et al., 2017 | Prevalence of burnout in health professionals working in palliative care: a systematic review. | Europe and other | Systematic review | 8 | Physicians, nurses, physiotherapists, psychologists, social workers and occupational therapists | Burnout. Maslach Burnout Inventory (MBI), MBI Human Services Survey | Burnout prevalence was 17.3% (based on three studies; no meta-analysis). Subscale prevalence rates were: Personal Accomplishment 19.5%, Emotional Exhaustion 17.5%, and Depersonalisation 6.5%. Among palliative care professionals, social workers showed the highest burnout (27%, one study), followed by nurses (18.6%) and physicians (15.1%). Burnout was highest in home care settings (19.6%) and lowest in hospices (14.2%). | High |
| **57** | Domagala et al., 2019 | Satisfaction of physicians working in hospitals within the European Union: state of the evidence based on systematic review. | Europe and other | Systematic review and meta-analysis | 61 | Physicians. Hospital | Job satisfaction. Job Diagnostic Survey (JDS); Copenhagen Psychosocial Questionnaire (COPSOQ); Leiden Quality of Work Questionnaire (scale for JS); Minnesota Job Satisfaction Questionnaire | The level of physician satisfaction in Europe is moderate. Estimated weighted percentage of satisfied physicians was 55.3% (95%CI 48.2-62.4), high heterogeneity (I2=99.08). Percentage of satisfied physicians varied from 21% to 95.5%. Greater proportions of women in a sample were associated with lower levels of satisfaction. | High |
| **59** | Pang et al., 2021 | Is urologist burnout different on the other side of the pond? A European perspective. | Europe and other | Literature review | 7 | Urologists. Hospital | Burnout. Maslach Burnout Inventory (MBI), Abbreviated MBI (aMBI) | Previous research indicated that urologists have the highest levels of burnout among physicians. While the rates vary (9.3-68%), they indicate that burnout is prevalent within urology. Age, higher responsibility (e.g. UK | Low |

| | | | | | | | | | |
|---|---|---|---|---|---|---|---|---|---|
| | | | | | | | | consultants), and greater administrative workload were associated with higher burnout. Structured mentorship programs helped to reduce burnout. | |
| **61** | Ungureanu et al., 2020 | The geriatric workforce in Romania: the need to improve data and management. | Europe: Romania | Rapid scoping review | 2 | HCWs. Geriatric health | Burnout. N/A | Both studies highlighted high levels of burnout among Romanian geriatric HCWs. Studies reported high levels of emotional exhaustion, depersonalization, and reduced personal accomplishment. Perceived job demands were correlated with emotional exhaustion and depersonalization | Low |
| **63** | Krepia et al., 2023 | Job Satisfaction of Nurses: A Literature Review. | Europe and other | Narrative review | Not specified | Nurses. Hospitals and clinical environments | Job satisfaction. N/A | Job satisfaction can be affected by financial compensation, working environment conditions, relations with colleagues and administration, and the nature of the job itself. | Low |
| **64** | Xie et al., 2021 | The prevalence of compassion satisfaction and compassion fatigue among nurses: A systematic review and meta-analysis. | Europe and other | Systematic review and meta-analysis | 79 | Nurses. Hospital and clinical environments | Compassion satisfaction, burnout, and secondary traumatic stress. Professional Quality of Life Scale. | The study found moderate levels of compassion satisfaction and compassion fatigue. For Europe, the compassion satisfaction mean score was of 32, the mean burnout score was of 24 and the secondary traumatic stress score was of 20. European nurses had one of the lowest burnout and secondary traumatic stress scores. | High |
| **65** | Poon et al., 2022 | A global overview of healthcare workers' turnover intention amid COVID-19 pandemic: a systematic review with future directions. | Europe and other | Systematic review | 43 | Nurses, physicians, allied health workers, healthcare administrative or management staff | Turnover intention. Turnover Intention Scale (TIS-6) and other self-report instruments. | Fear of COVID-19 exposure, psychological stress, adverse working conditions, socio-demographic factors, and organizational support were associated with turnover intention. | High |
| **66** | Carton & Hupcey, 2014 | The Forgotten Mourners. | Europe and other | Systematic review | 12 | Health care providers, nurses, resident physicians. Paediatric, oncology, and palliative care settings | Grief, burnout, perceived stress, professional distress, and coping mechanisms | Grief among health care providers is prevalent but often unrecognized, leading to burnout, compassion fatigue, and professional distress. Various interventions were assessed, but most studies lacked rigorous experimental designs. | Moderate |

| | | | | | | | | | |
|---|---|---|---|---|---|---|---|---|---|
| **67** | Matsuishi et al., 2021 | Severity and prevalence of burnout syndrome in paediatric intensive care nurses: A systematic review. | Europe and other | Systematic review | 6 | Nurses. Paediatric intensive care unit (PICU). | Burnout. Maslach Burnout Inventory (MBI), Abbreviated MBI (aMBI), Occupational Burnout Inventory (OBI), Professional Quality of Life Scale (ProQoL). | Prevalence of burnout in PICU nurses ranged between 42% and 77%. Severity appeared higher for PICU compared to other paediatric and adult units. | Moderate |
| **68** | Patterson et al., 2011 | Systematic review of the links between human resource management practices and performance. | Europe and other | Systematic review | 10 | HCWs. Any | Job satisfaction, stress, burnout, turnover intention, psychological well-being | HRM practices like team working and job design changes, showed mixed effects on HCWs outcomes. The impact of interventions varied by settings and implementations with no consistently positive results across all studies. | Moderate |
| **70** | Ho & Chiang, 2015 | A meta-ethnography of the acculturation and socialization experiences of migrant care workers. | Europe and other | Meta-ethnography | 25 | Migrant HCWs. Healthcare sector. | Acculturation and socialization. Qualitative methods. | Migration is driven by optimism but often confronted with barriers. Adaptation is shaped by personal resources and environmental stressors. Migrant nurses found to be especially vulnerable. | Moderate |
| **71** | Spector et al., 2014 | Nurse exposure to physical and nonphysical violence, bullying, and sexual harassment: A quantitative review. | Europe and other | Quantitative review | 136 | Nurses. Emergency, geriatric, hospital, and psychiatric facilities | Physical violence, non-physical violence, bullying, sexual harassment, and injury. | In Europe, nurses experience significant workplace violence, with 35% reporting physical violence and 60% experiencing nonphysical violence. While bullying rates (8.8%) were the lowest among all regions studied, sexual harassment (16.2%) remained a concern. | Low |
| **72** | Drudy et al., 2014 | The impact of organisational change in the NHS on staff and patients: a literature review with a focus on mental health. | Europe: UK | Literature review | 49 | HCWs. Mental health | Impacts caused by organisational change | Organisational and structural challenges impacted workforce mental health and service delivery. | Moderate |
| **73** | Dodd et al., 2017 | PTSD, available support and development of services in the UK Ambulance Service. | Europe: UK | Narrative review | Not specified | Ambulance staff. Ambulance and pre-hospital care | PTSD, anxiety, depression. | PTSD, anxiety, and depression are common among ambulance personnel due to frequent exposure to trauma and work-related stress. 92% experienced poor mental health, and 62% had receive treatment for mental health problem. | Low |

| | | | | | | | | | |
|---|---|---|---|---|---|---|---|---|---|
| **75** | Lea, Corlett, and Rodgers, 2012 | Workload and its impact on community pharmacists' job satisfaction and stress: a review of the literature. | Europe: UK | Literature review | 13 | Pharmacists. Community pharmacy | Workload, job satisfaction and stress. | Pharmacists spend most of their working day in dispensing activities. Increased workload contributes to higher stress and lower job satisfaction. Pharmacists report a perception of increasing workload over time. Workload pressures are lading to patient safety concerns. | Moderate |
| **76** | Sirois & Owens, 2021 | COVID-19: nurses' distress higher than for colleagues: Studies find mental health effects could last for up to three years. | Europe and other | Rapid systematic review | 139 | HCWs. Healthcare sector during infectious outbreaks. | Anxiety, depression, PTSD, stress and burnout. Perceived Stress Scale (PSS-10), Depression, Anxiety, Stress Scales (DASS-21), Generalized Anxiety Disorder Scale (GAD-7), State-Trait Anxiety Inventory (STAI), Hospital Anxiety and Depression Scale (HADS), Patient Health Questionnaire (PHQ-9), Beck Depression Inventory (BDI-II), Impact of Event Scale (IES-R), Maslach Burnout Inventory (MBI), General Health Questionnaire (GHQ-12), Symptom Checklist-90-R (SCL-90-R) | Risk factors: being female, younger age, nurse role, stigma, maladaptive coping, exposure to infected patients, quarantine. Protective factors: social support, perceived control, positive work attitudes, sufficient information, PPE/resources/training. Distress can persist up to 3 years post-outbreak | High |
| **77** | Fountouki et al., 2011 | Nursing staff under heavy stress: focus on Greece A critical review. | Europe and other | Critical review | 26 | Nurses. Hospital and clinical environments | Stress, anxiety, burnout, depression, job dissatisfaction, and PTSD | Major stress factors included poor communication with superiors, dissatisfaction with psychosocial work environment, insufficient work resources, shift work challenges, verbal abuse, mobbing, poor organizational support. | Low |
| **78** | Pich et al., 2010 | Patient-related violence against emergency department nurses. | Europe and other | Literature review | 53 | Nurses. Emphasis on emergency department | Impact of violence: retention, psychological well-being, and quality of care. | Patient-related violence against nurses, especially in EDs, is frequent and often normalized; chronic under-reporting persists; risk factors include substance abuse, mental illness, long | Moderate |

| | | | | | | | | | |
|---|---|---|---|---|---|---|---|---|---|
| | | | | | | | | waiting times, night shifts, and environmental design. Violence negatively affects nurse retention, psychological well-being, and quality of care. | |
| **79** | Lee et al., 2013 | Correlates of physician burnout across regions and specialties: a meta-analysis. | Europe and other | Meta-analysis | 65 | Physicians. Hospital and clinical environments | Burnout, emotional exhaustion and depersonalization. Maslach Burnout Inventory (MBI) | The findings indicate that workload, organizational constraints, work-life conflict, and contributors to poor mental health were strongly associated with burnout, whereas autonomy, quality and safety culture, and positive work attitudes were protective. | High |
| **80** | Burton et al., 2016 | How Effective are Mindfulness-Based Interventions for Reducing Stress Among Healthcare Professionals? A Systematic Review and Meta-Analysis. | Europe and other | Systematic review and meta-analysis | 9 | Nurses, midwives, primary care clinicians, mental health professionals, and other health care personnel. Hospital and clinical environments | Reduction in stress levels and improvements in mindfulness, well-being, self-compassion, burnout, anxiety, depression and overall psychological distress. Perceived Stress Scale (PSS), Mental Health Professionals Stres Scale (MHPSS), Depression Anxiety Stress Scale (DASS), Survey of Recent Life Experiences (SRLE), Visual Analogue Scale (VAS), Mindfulness Attention Awareness Scale (MAAS), Five Facet Mindfulness Questionnaire (FFMQ), Toronto Mindfulness Scale (TMS). | Mindfulness-Based Interventions (MBIs) showed moderate effectiveness in reducing stress among healthcare professionals. Most studies reported significant stress reduction post-intervention. However, methodological weaknesses (e.g., small sample sizes, lack of theoretical frameworks) and potential publication bias (file drawer problem) were noted. | High |
| **82** | Smedra et al., 2021 | Suicide committed by a paramedic using a cocktail of drugs: Morphine, etomidate, diazepam and rocuronium. Case report and review of literature. | Europe and other | Case report and literature review | Not specified | Paramedics and other HCWs. Healthcare sector | Suicide | Healthcare professionals have elevated suicide rates compared to the general population, largely due to occupational stress, mental health disorders, and easy access to potent drugs, leading to frequent use of poisoning as a method. Stigma and undertreatment of mental illness are common among this group. Prevention efforts should | Low |

| | | | | | | | | | |
|---|---|---|---|---|---|---|---|---|---|
| | | | | | | | | prioritize mental health support, destigmatization, and limiting access to lethal means. | |
| **83** | Ball et al., 2015 | A UK Review Corroborates the Negative Consequences of 12-Hour Workdays. | Europe and other | Research synthesis | 26 | Nurses. Acute hospital setting | Fatigue, stress, burnout, performance, quality of care, and job satisfaction | 12-hour shifts are increasing common across healthcare settings. These shifts are associated with increased fatigue, reduced sleep, higher rates of job dissatisfaction, poorer performance, and greater risk to patient safety. However, some nurses prefer 12-hour shifts for better work-life balance. | Moderate |
| **84** | Kunzler et al., 2020 | Psychological interventions to foster resilience in healthcare professionals | Europe and other | Cochrane systematic review | 44 | Nurses, physicians, hospital personnel and allied healthcare staff. Hospital and clinical environments | Resilience, well-being, anxiety, depression, stress. | Moderate improvements in resilience (SMD=0.45), and small improvements in depression (SMD=-0.29) and stress (SMD=-0.61) post-interventions. Little or no effect on anxiety or overall well-being. Interventions were face-to-face and of high intensity. Most effects measured in the short term. | High |
| **85** | Zimmermann et al., 2024 | Suicide rates among physicians compared with the general population in studies from 20 countries: Gender stratified systematic review and meta-analysis. | Europe and other | Systematic review and meta-analysis | 39 | Physicians. N/A | Suicide | Female physicians had higher suicide rate compared with the general population (RR=1.76), while male physicians did not. Suicide rates have decreased over time for both genders but risk remains elevated for female physicians, and male physicians have higher suicide risk compared with other professional groups. | High |
| **86** | Ma et al., 2023 | The prevalence, related factors and interventions of oncology nurses' burnout in different continents: A systematic review and meta-analysis. | Europe and other | Systematic review and meta-analysis | 20 | Nurses. Oncology wards and cancer treatment centres | Burnout. Maslach Bunout Inventory (MBI) | Overall prevalence of high burnout symptoms: EE 36.4%, DP 28.3%, PA 28.7%. Factors influencing burnout differ by region (personal in Asia, work related in Europe). | High |
| **87** | Ge et al., 2023 | Global prevalence of nursing burnout syndrome and temporal trends for | Europe and other | Meta-analysis | 94 | Nurses. Hospital and clinical environments | Burnout. Maslach Burnout Inventory (MBI), Professional Quality of Life | Global prevalence of nursing burnout was 30%. Pooled prevalence of burnout in Europe was 32%. Meta-regression | High |

| | | | | | | | | | |
|---|---|---|---|---|---|---|---|---|---|
| | | the last 10 years: A meta-analysis of 94 studies covering over 30 countries. | | | | | (ProQoL), Copenhagen Burnout Inventory (CBI), Spanish Burnout Inventory (SBI), Well-Being Index (WBI) | showed a significant increase in burnout prevalence over the past 10 years, particularly in Europe. | |
| **88** | Tamming et al., 2023 | Individual-level interventions for reducing occupational stress in healthcare workers | Europe and other | Cochrane systematic review | 117 | HCWs. Hospital settings, residential care, emergency care, mixed settings. | Stress and burnout. Maslach Burnout Inventory (MBI), Perceived Stress Scale (PSS), Depression Anxiety Stress Scale (DASS). | Interventions that focused on the experience of stress (SMD=-0.37 to -0.43) or away from it (SMD=-0.41 to -0.55) may reduce stress symptoms in the short to medium term. A combination of interventions may be especially effective (SMD=-0.48 to -0.67). | High |

[a]Quality classification was assessed using the Joanna Briggs' Institute Critical Appraisal Tool for Systematic Reviews. Each of the eleven criteria was scored as 1 ("yes") or 0 ("no"/"maybe"). The quality of studies was categorised as low (0-5), medium (6-8) or high (9-11) quality.

**Supplementary Table 3. Summary of studies included by research question and ecological domain**

| Section | Ecological level | Findings |
| --- | --- | --- |
| Prevalence of psychological distress among HCWs | - | Twenty reviews were included in this section [3, 20,26, 27,31,34, 35, 43,45, 49,56, 59, 64,67, 73, 75, 78, 85, 86,87], nine of which focused exclusively on European studies or provided Europe specific estimates, and 11 included European and non-European countries. |
| Factors that exacerbate or mitigate risks to HCW psychological distress and retention, including inequalities that may affect subgroups of HCWs | Sociodemographic factors | A total of fifteen reviews examined sociodemographic, psychosocial, and health-related risk factors [1, 3, 7, 11, 18, 21, 25, 45, 50, 59, 65, 72, 76, 79, 87]. These reviews included a range of HCW populations such as nurses, physicians, pharmacists, occupational therapists, maternity staff, forensic professionals, urologists, mental health staff, allied healthcare, and health support workers, as well as broader HCW groups. Most reviews drew on data from both European and international settings, only three reviews focused specifically on UK-based findings [7, 25, 72]. |
| | Psychosocial factors | Four reviews explored psychosocial factors (11, 21, 76, 79). The reviews included data from HCWs (any) (n=1), physicians (n=1), nurses (n=1), and occupational therapists (n=1). All the reviews included data from various regions, including Europe. |
| | Health factors | Three reviews reported on physical and mental health related factors (3, 21, 65). Each reported findings from maternity staff, nurses, and HCWs (any) and all of them included information from Europe and other regions. |
| | Professional factors | Nineteen reviews reported findings on professional factors [1, 11, 17, 18, 27, 32, 34, 35, 36, 43, 56, 59, 63, 64, 67, 72, 73, 78, 87]. Most reviews included nurses (n=10), and the other reviews included HCWs (any) (n=3), occupational therapists (n=1), forensic professionals (n=1), oncologists (n=1), physicians (n=1), urologists (n=1), ambulance staff (n=1), and mental health staff (n=1). Three studies included in this section report results only from Europe (UK), all other studies included European data and data from other regions. |
| | Work-related psychosocial factors | Sixteen reviews reported findings on work-related psychosocial factors [1, 2, 3, 7, 11, 16, 17, 19, 21, 22, 29, 32, 65, 67, 76, 78]. Reviews covered a range of HCW occupations including HCWs (mixed) (n=3), nurses (n=8), mental health professionals (n=1), maternity staff (n=1), pharmacists (n=1), and occupational therapists (n=1). Three reviews reported results exclusive to European countries [7, 22, 32], |

| | | |
|---|---|---|
| | | with all other reporting on a mix of European and international studies. |
| | Financial factors | Four reviews reported on financial factors and their role on occupational outcomes [1, 33, 63, 77]. All four reviews were conducted in specific occupational groups: three reviews focused on nurses [1, 77, 63] and one review focused on midwives [33]. Although one review focused broadly on the implications of their review findings on nurses in Greece [77], none of the reviews had a specific focus on the European context. |
| | Organizational factors | Thirty-one reviews reported findings on organizational factors [1, 3, 6, 7, 11, 17, 18, 19, 21, 22, 27, 33, 39, 44, 74, 86, 43, 45, 65, 76, 79, 53, 68, 83, 72, 49, 40, 77, 61, 59, 63]. Reviews covered a range of HCW occupations including HCWs (mixed) (n=9), nurses (n=12), physicians (n=3), pharmacists, occupational therapists, mental health staff, maternity staff, ambulance/emergency response staff, forensic professionals, frontline HCWs, and geriatric HCWs (all n=1). Eight reviews reported results exclusive to European countries [7, 19, 22, 39, 43, 40, 59], with all others reporting on a mix of European and international studies. |
| | Political factors | Three reviews reported on political factors impacting on occupational outcomes [1, 29, 32]. All three reviews focused on migrant nurses. One review [32] specifically reported evidence relating to migrant nurses in England, with the two other reviews including both European and international studies. |
| Evidence on interventions to prevent or treat mental health issues in HCWs | - | Twelve reviews examined various interventions to reduce or treat mental health issues in HCWs [10, 13, 15, 24, 42, 48, 54, 66, 68, 80, 84, 86]. Thee revies [48, 68, 54] reported findings only from Europe. |

*Numbers in table refer to paper ID in Supplementary Table 2.

**Supplementary Figure 1. Flow diagram of study selection for the umbrella review**

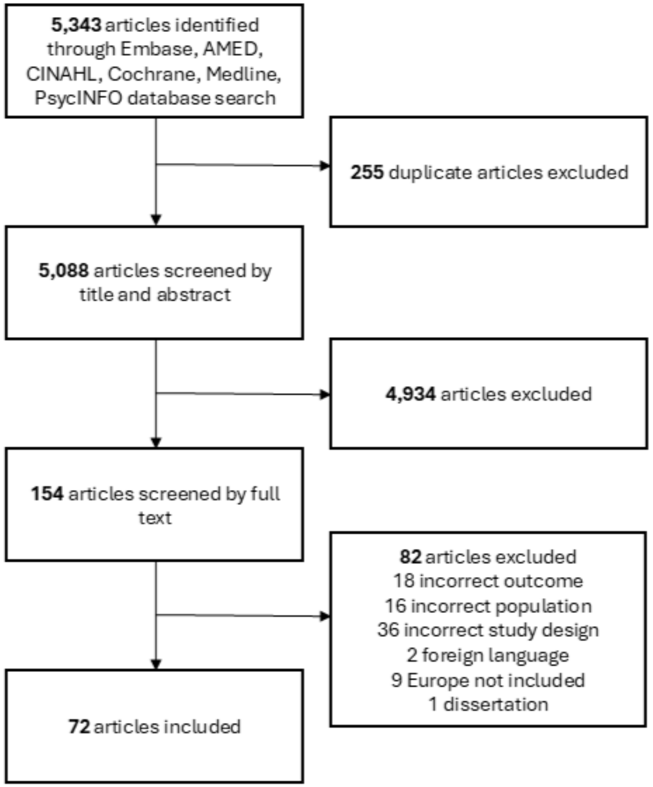


**Figure 1**. Study selection
AMED: Allied and Complementary Medicine Database
CINAHL: Cumulative Index to Nursing and Allied Health Literature
